# Mechanistic insights into hydroacylation with non-chelating aldehydes†
†Electronic supplementary information (ESI) available: Materials and methods, reaction procedures, characterization data. CCDC 1012849. For ESI and crystallographic data in CIF or other electronic format see DOI: 10.1039/c4sc02026j



**DOI:** 10.1039/c4sc02026j

**Published:** 2014-09-22

**Authors:** Stephen K. Murphy, Achim Bruch, Vy M. Dong

**Affiliations:** a Department of Chemistry , University of California , Irvine , California 92697-2025 , USA . Email: dongv@uci.edu; b Department of Chemistry , University of Toronto , 80 St. George Street , Toronto , Ontario M5S 3H6 , Canada

## Abstract

The combination of a small-bite-angle diphosphine bis(dicyclohexylphosphino)methane (dcpm) and [Rh(cod)OMe]_2_ catalyses the hydroacylation of 2-vinylphenols with a wide range of non-chelating aldehydes. Here we present a detailed experimental study that elucidates the factors contributing to the broad aldehyde scope and high reactivity. A variety of catalytically relevant intermediates were isolated and a [Rh(dcpm)(vinylphenolate)] complex was identified as the major catalytically relevant species. A variety of off-cycle intermediates were also identified that can re-enter the catalytic cycle by substrate- or 1,5-cyclooctadiene-mediated pathways. Saturation kinetics with respect to the 2-vinylphenol were observed, and this may contribute to the high selectivity for hydroacylation over aldehyde decarbonylation. A series of deuterium labelling experiments and Hammett studies support the oxidative addition of Rh to the aldehyde C–H bond as an irreversible and turnover-limiting step. The small bite angle of dcpm is crucial for lowering the barrier of this step and providing excellent reactivity with a variety of aldehydes.

## Abstract 2

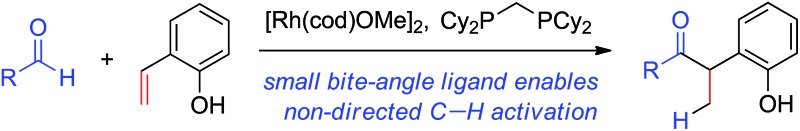
Rhodium catalysts with small-bite-angle diphosphines enable branched-selective hydroacylation of 2-vinylphenols *via* C–H activation of non-chelating aldehydes.

## Introduction

**Fig. 1:**
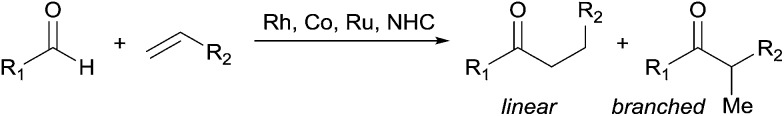
Intermolecular olefin hydroacylation.

Central challenges in Rh-catalysed olefin hydroacylation are to promote aldehyde C–H activation while preventing decarbonylation, and to control the regioselectivity of C–C bond formation (Fig. 1).^1^ Most intermolecular hydroacylations are inherently linear-selective and rely on chelating aldehydes such as salicylaldehydes,^2^ 2-aminobenzaldehydes,^3^ β-sulphur aldehydes,^4^ or 2-pyridylaldimines^5^ to promote C–H activation and suppress decarbonylation. Brookhart, Tanaka, and Jun have made breakthroughs in the field by developing linear-selective protocols with non-chelating aldehydes.^6^ Alternatively, Co catalysts,^7^ Ru hydrides^8^ and N-heterocyclic carbenes^9^ have emerged as promising catalysts for the linear-selective coupling of aldehydes with α,β-unsaturated carbonyl compounds, 1,3-dienes, cyclopropenes, and some styrenes. Developing methods that provide the alternative branched products without relying on chelating aldehydes would enable access to unique chemical space by hydroacylation.

**Fig. 2:**
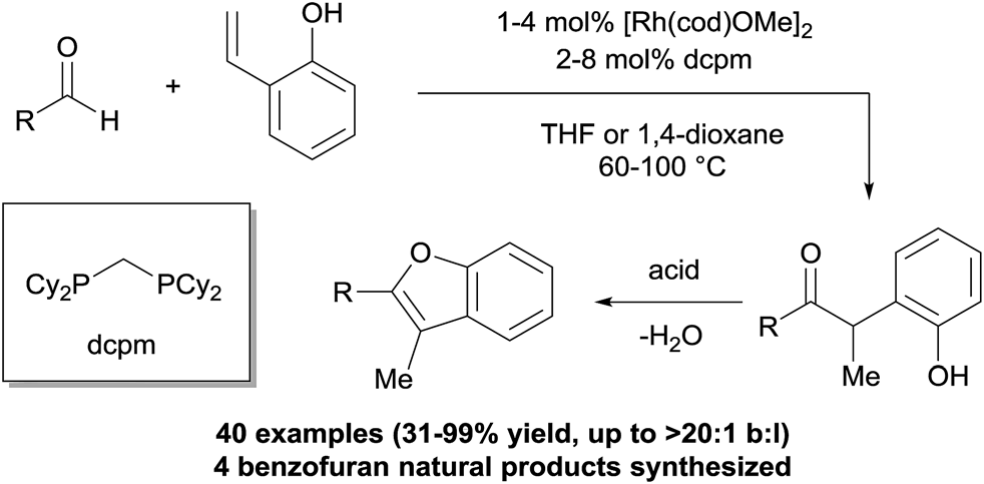
Intermolecular 2-vinylphenol hydroacylation and cyclocondensation to access benzofuran natural products.

We recently reported a strategy for branched-selective hydroacylation with an aldehyde scope that is among the most broad reported to date (Fig. 2).^10^ Alkyl, alkenyl, and aryl aldehydes were coupled with 2-vinylphenols in generally >20 : 1 branched-to-linear (b : l) selectivity and excellent yields. We hypothesized that the phenolic group on the olefin promotes the branched regioselectivity in analogy to our double-chelating hydroacylations with salicylaldehydes.^2c–e^ A [Rh(cod)OMe]_2_ catalyst in combination with a small-bite-angle diphosphine, bis(dicyclohexylphosphino)methane (dcpm), was crucial for reactivity. This method enabled short syntheses of four neolignan natural products using hydroacylation in combination with an acid-catalysed cyclocondensation. Herein we report a mechanistic study that sheds light on the catalyst resting states of this transformation, the rate-limiting step, and the properties of the substrates and ligands that enable reactivity with non-chelating aldehydes.

## Results and discussion

### A. Assignment of catalyst resting states

To identify potential reaction intermediates, we monitored the ^31^P NMR of a catalytic hydroacylation involving hydrocinnamaldehyde (**1**) and 4-nitro-2-vinylphenol (**2**) (Table 1). At 12% conversion, four different phosphorus-containing complexes (**4**, **5**, **6** and **7**) were detected in an approximately 3 : 3 : 1 : 1 ratio, respectively, based on integration of the ^31^P NMR signals. Over the course of catalysis, complexes **5** and **6** decreased in concentration while only complexes **4** and **7** remained at the end of the reaction.

**Table 1:** ^31^P NMR spectrum of a catalytic reaction at 12% conversion

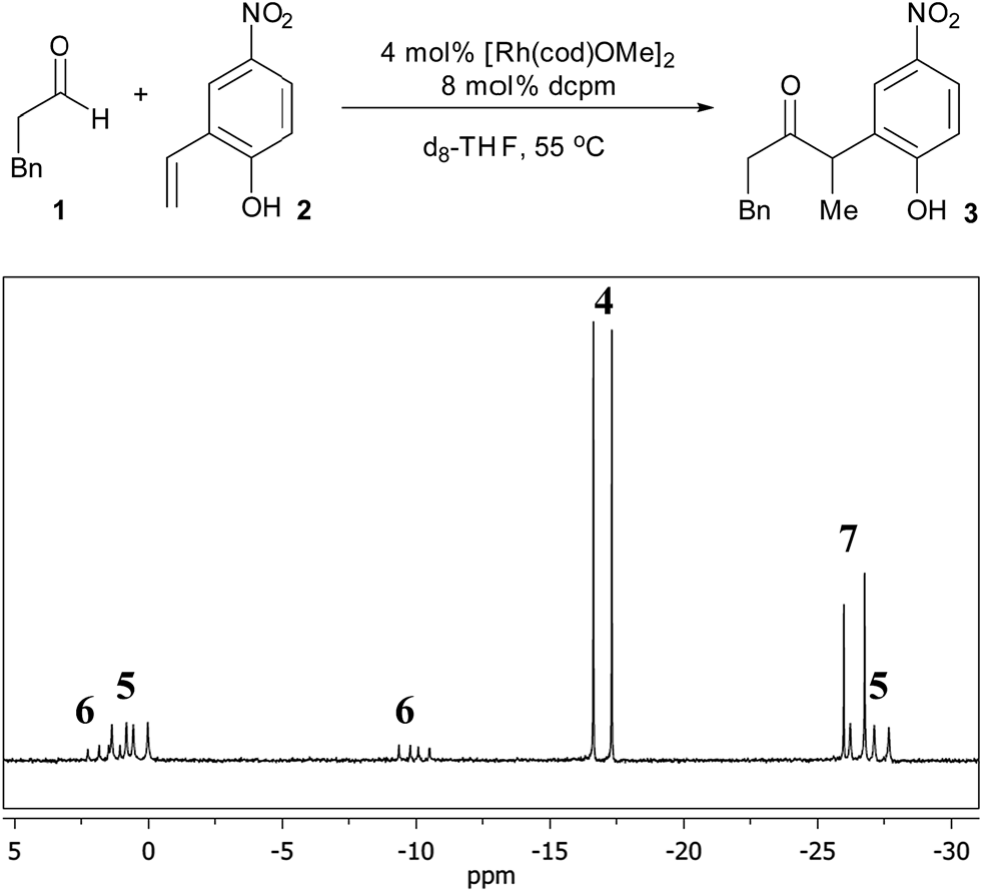			
Complex	*δ* (ppm)	*J*_Rh–P_ (Hz)	*J*_P–P_ (Hz)
**4**	–17.0	111	—
**5**	0.7, –26.9	125, 148	92
**6**	1.7, –9.9	126, 117	69
**7**	–26.4	126	—

**Fig. 3:**
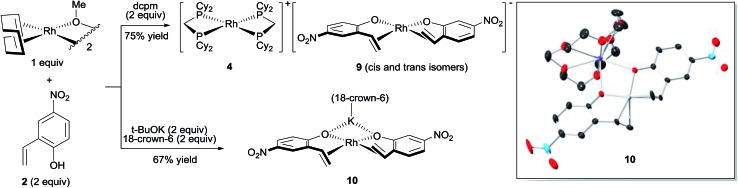
Synthesis of [Rh(vinylphenolate)_2_]^–^ complexes.

The major complex **4** was prepared by reacting [Rh(cod)OMe]_2_ with dcpm and 4-nitro-2-vinylphenol in a 1 : 2 : 2 molar ratio in THF (Fig. 3, top). A cationic [Rh(dcpm)_2_]^+^ fragment was identified by ESI^+^ MS and by comparison of the NMR data with that of [Rh(dcpm)_2_]BF_4_ (**8**). The synthesis of **4** was accompanied by quantitative formation of 1,5-cyclooctadiene, MeOH and another Rh complex **9**, containing a coordinated vinylphenolate anion as judged by ^1^H NMR analysis. The aromatic protons of the vinylphenolate were shifted upfield and the vinylic peaks appeared between 2 and 4 ppm—within the range of coordinated olefins. The ^13^C NMR showed coupling between the vinylic carbon atoms and the Rh center (*J*_Rh–C_ = 11 and 15 Hz). Based on this data and the ESI^–^ MS, we assigned this complex as an anionic 2 : 1 vinylphenolate-to-Rh complex **9**. To support our assignment of anion **9**, the [(18-crown-6)K][Rh(nitrovinylphenolate)_2_] salt **10** was synthesized by treating two equivalents of 4-nitro-2-vinylphenol with [Rh(cod)OMe]_2_, *t*-BuOK and 18-crown-6 (Fig. 3, bottom). The solid state structure of salt **10** (Fig. 3, right) contains two vinylphenolate anions bound to a Rh atom in a *cis* orientation, where the phenolate oxygen atoms bind to the potassium centre. Only small differences in chemical shift were noted between **9** and **10**, which are likely due to potassium coordination in **10**. Additionally, small shoulder peaks were noted in the ^1^H NMR spectrum of **9**, which are likely a result of geometrical isomers.

Given that the double salt of **4** and **9** accounts for nearly 60% of the Rh during catalysis, the reactivity of these species strongly correlates with the activity of the overall catalyst system. We thus decided to examine the reactivity of each ion independently by studying complexes **8** ([Rh(dcpm)_2_]BF_4_) and **10** [(18-crown-6)K][Rh(nitrovinylphenolate)_2_] (Table 2). While [Rh(diphosphine)_2_]^+^ complexes catalyse the cyclization of 4-pentenals at elevated temperatures,^11^ complex **8** did not catalyse vinylphenol hydroacylation even in the presence of base (*t*-BuOK, entries 2 and 3). Salt **10** also did not catalyse hydroacylation (entry 4), but it could be activated by dcpm to give an initial turnover frequency (TOF_init_) four times lower than the optimized reaction (entries 1 and 5, 15 h^–1^ *versus* 4 h^–1^, respectively). Although this rate is low, the observation of catalysis supports the intermediacy of phosphine-ligated species and suggests that **4** and **9** are not directly on the catalytic cycle. The combination of **8**, **10**, and 1,5-cyclooctadiene resulted in slow catalysis (entries 6 and 7, approx. 50% conv. after 24 hours). Apparently, the 1,5-cyclooctadiene converts the double salt back to a catalytically active species, although this effect is likely smaller than the inhibitory effect of 1,5-cyclooctadiene that occurs due to binding of the active catalyst (*vide infra*).

**Table 2:** Hydroacylation with various Rh complexes

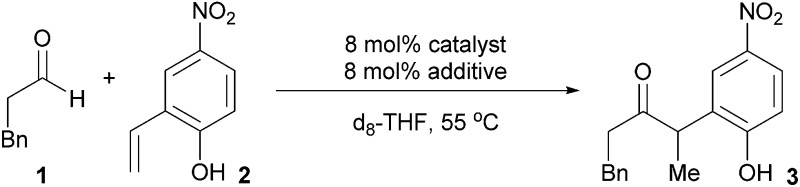				
Entry	Catalyst	Additive	Yield^*b*^ (%)	Time (h)
1	[Rh(cod)OMe]_2_ ^*a*^	dcpm	>95	2
2	**8**	—	n.r.	—
3	**8**	*t*-BuOK	n.r.	—
4	**10**	—	n.r.	—
5	**10**	dcpm	80	12
6	**8**/**10**	—	n.r.	—
7	**8**/**10**	cod	50	24

^*a*^4 mol% catalyst.

^*b*^n.r. = no reaction.

**Fig. 4:**
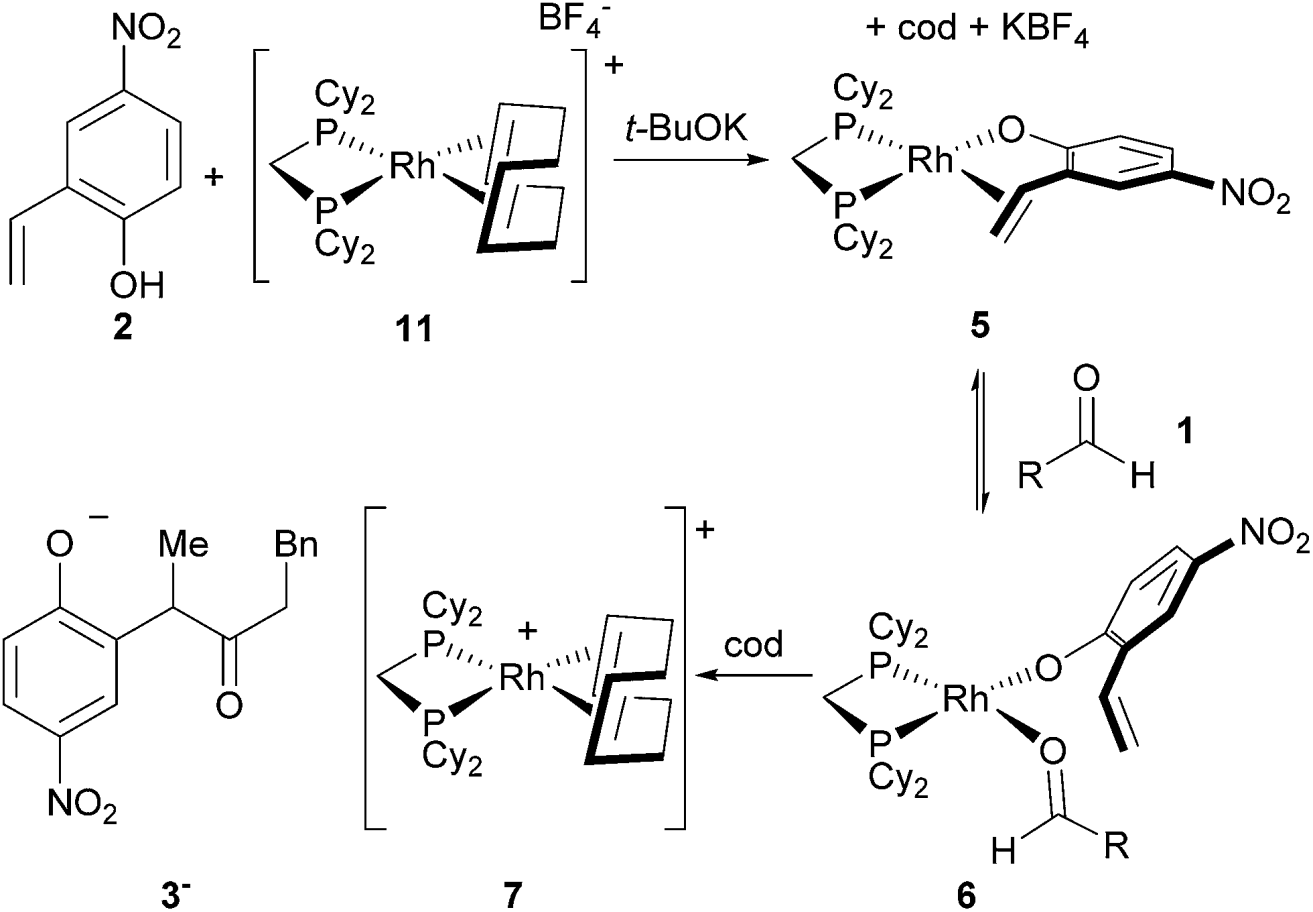
Formation of the olefin-bound complex and its behaviour in the presence of excess aldehyde (R = CH_2_Bn).

Complex **7** is the next most abundant Rh complex during catalysis, and was identified as a cationic [Rh(dcpm)(cod)]^+^ fragment by comparison of the ^31^P NMR spectrum to that of [Rh(dcpm)(cod)]BF_4_ (**11**). This latter complex is a kinetically competent catalyst in the presence of *t*-BuOK and provides a similar initial turnover frequency to the optimized conditions (18 h^–1^ *versus* 15 h^–1^, respectively).^12^ When a suspension of 4-nitro-2-vinylphenol and *t*-BuOK was vigorously stirred with complex **11**, a nearly quantitative exchange reaction occurred to generate substrate-bound **5** (Fig. 4). In the presence of excess aldehyde, the olefin ligand of complex **5** exchanges with the aldehyde to generate intermediate **6**, which is observed during catalysis. Complex **6** is only observed transiently and undergoes hydroacylation and a substitution reaction at room temperature to form cation **7** and anion **3^–^**, which aggregates with excess phenols as judged by ESI^–^ MS.

Our investigation of catalyst resting states reveals that **5** and **6** are directly on the catalytic cycle, while a variety of off-cycle intermediates can re-enter the cycle *via* pathways mediated by 1,5-cyclooctadiene or 2-vinylphenol. The strong coordination of the 2-vinylphenol compared to the aldehyde is likely a key to promoting hydroacylation over competitive decarbonylation. Of the observed Rh complexes, oxidative addition would only be possible from **6**, which already contains the O-coordinated 2-vinylphenolate moiety. Formation of the acyl-Rh-hydride would likely be followed by rapid coordination of the olefin to generate a coordinatively saturated intermediate that is stable towards decarbonylation,^13^ thus providing the desired chemoselectivity.

### B. Kinetic analysis using *in situ* NMR monitoring of reaction progress

**Fig. 5:**
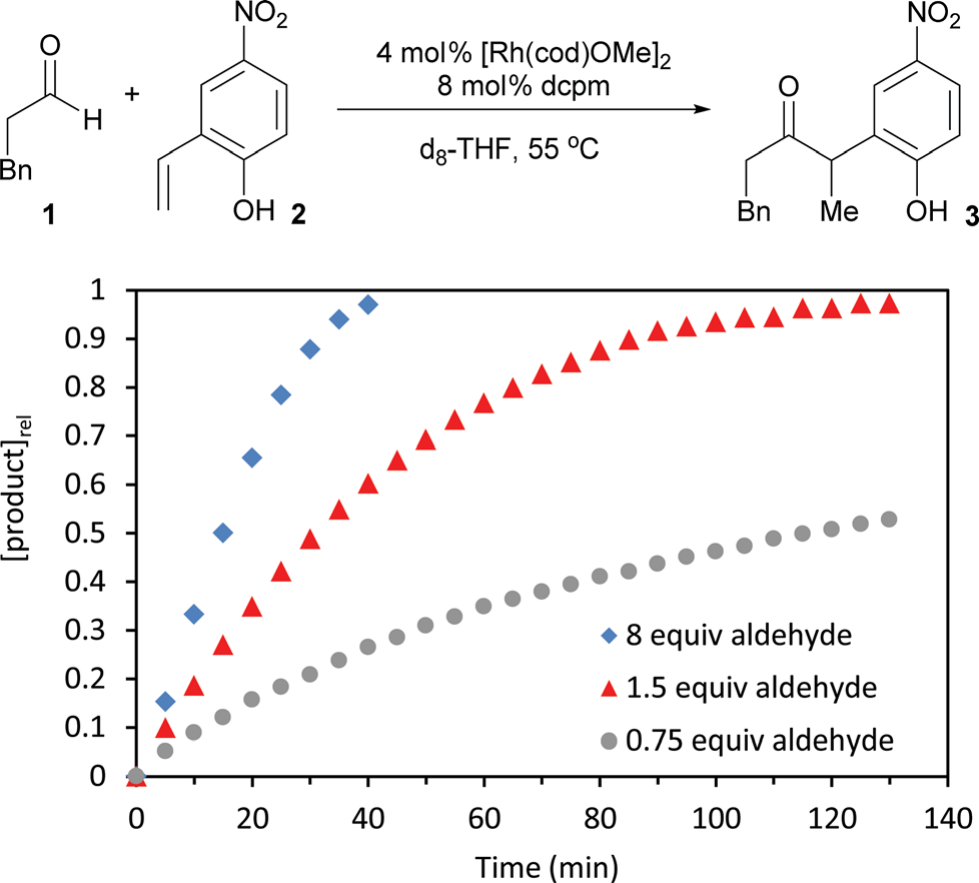
Kinetic data for hydroacylation with various amounts of hydrocinnamaldehyde.

**Fig. 6:**
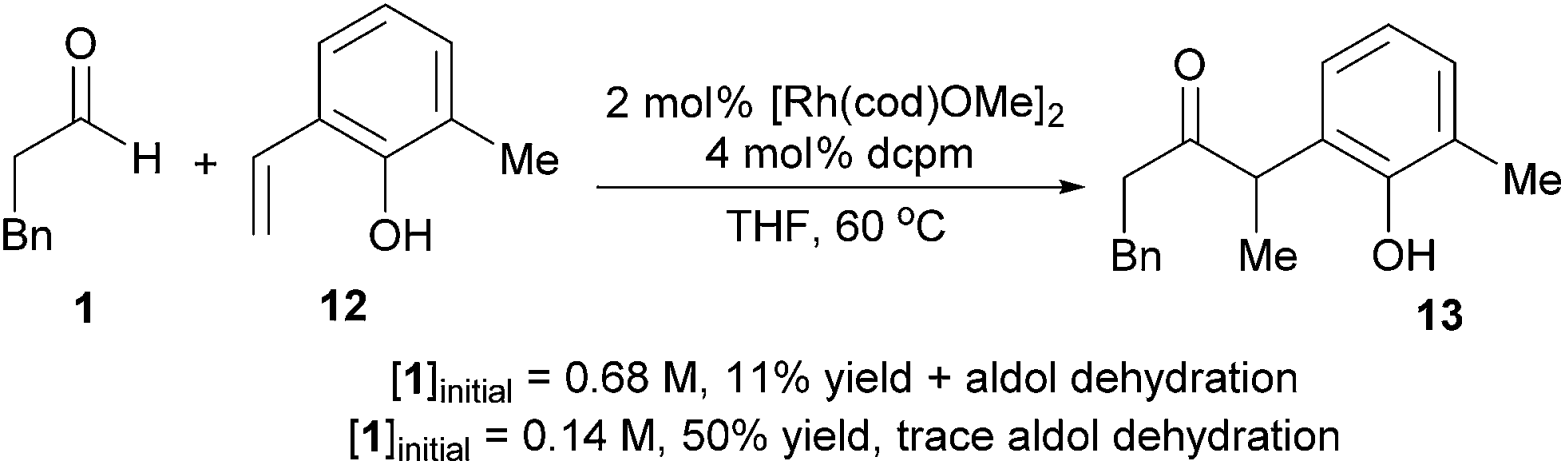
Concentration-dependent chemoselectivity for hydroacylation over aldol dehydration.

To identify kinetic parameters, we studied the hydroacylation of 4-nitro-2-vinylphenol under catalytic conditions with various amounts of hydrocinnamaldehyde (Fig. 5). The coupling reaction with 1.5 equivalents of hydrocinnamaldehyde follows a curved reaction profile with an initial turnover frequency of 15 h^–1^ and no induction period. Decreasing the aldehyde concentration by half decreases the initial turnover frequency by half (8 h^–1^), a result that suggests a first order dependence of the rate on aldehyde concentration. Using a large excess of aldehyde (8 equiv) yields a reaction profile that is linear up to 80% conversion with an initial turnover frequency of only 23 h^–1^. Analysing the ^31^P spectrum under these highly concentrated conditions reveals that the equilibrium between **5** and **6** is completely shifted toward **6**. The formation of **6**, which contains both a coordinated aldehyde and phenolate, leads to saturation kinetics with respect to both substrates. Under typical catalytic conditions, however, this kinetic data and the assignment of catalyst resting states supports a first order dependence of the rate on aldehyde concentration and a zeroth order dependence on 2-vinylphenol concentration. These results corroborate the concentration-dependent selectivity for hydroacylation over competitive aldol dehydration (Fig. 6).

The substrate 6-methyl-2-vinylphenol (**12**) undergoes slow hydroacylation due to its steric bulk, which allows the aldehyde to be consumed by dimerization. However, greater chemoselectivity for hydroacylation over aldol dehydration was observed upon fivefold dilution. Diluting the reaction slows down aldol dehydration (second order in aldehyde) more dramatically than vinylphenol hydroacylation (first order in aldehyde, zeroth order in vinylphenol).

### C. Deuterium labelling studies

We probed the turnover-limiting step of the reaction by tracking the deuterium label for hydroacylation with deuterated 2-naphthaldehyde (Table 3, entry 1). This substrate coupled with 4-nitro-2-vinylphenol in 78% yield and 12 : 1 b : l selectivity. ^1^H and ^2^D NMR analysis revealed that deuterium was incorporated into only the β-positions of the resulting ketones. Analysis of the products by mass spectrometry revealed that they were exclusively monodeuterated. Analogous results were obtained for the reaction of deuterated hydrocinnamaldehyde with 4-nitro-2-vinylphenol (Table 3, entry 2). The lack of deuterium scrambling and multiply-deuterated products rules out reductive elimination as the turnover-limiting step and suggests that at least one of the earlier steps in the catalytic cycle is irreversible.

**Table 3:** Deuterium labelling studies (olefin = 4-nitro-2-vinylphenol)^*a*^ ^,^ ^*b*^ ^,^ ^*c*^

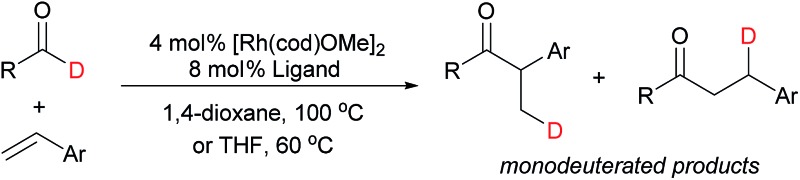					
Entry	Ligand^*d*^	R	Time (h)	Yield (%)	b : l
1	dcpm	2-nap	24	78	12 : 1
2	dcpm	CH_2_Bn	2	90	>20 : 1
3	dcpe	CH_2_Bn	12	45	>20 : 1
4	dcpp	CH_2_Bn	12	41	>20 : 1
5	dcpb	CH_2_Bn	12	35	>20 : 1

^*a*^b : l ratios were determined by NMR analysis of the crude reaction mixtures.

^*b*^Entry 1: 1,4-dioxane, 100 °C. Entries 2–5: THF, 60 °C.

^*c*^Deuterium content measured by ^1^H and ^2^D NMR, and ESI MS.

^*d*^dcpe = 1,2-bis(dicyclohexylphosphino)ethane; dcpp = 1,3-bis(dicyclohexylphosphino)propane; dcpb = 1,4-bis(dicyclohexylphosphino)butane.

This observation contrasts almost all other olefin hydroacylation studies where extensive deuterium-scrambling is observed as a result of rate-limiting reductive elimination.^14,15^ When larger-bite-angle diphosphines were used in the test for deuterium scrambling (Table 3, entries 3–5), the reaction rates were significantly reduced, deuterium scrambling still did not occur, and only monodeuterated products were identified. These results suggest that reductive elimination is not rate-determining for this substrate combination, regardless of ligand bite angle. Accordingly, the fact that smaller-bite-angle diphosphines provide faster rates can be attributed to their abilities to promote earlier steps in the catalytic cycle (*vide infra*).

To further probe the turnover-limiting step, we measured kinetic isotope effects (KIE) *via* intermolecular competition experiments of deuterated aldehydes with their protio analogues.^16^ With 4-nitro-2-vinylphenol as the coupling partner, a KIE of 2.5 ± 0.2 was observed for the reaction with hydrocinnamaldehyde and 2.4 ± 0.2 for the reaction with 2-naphthaldehyde. The similar magnitudes of the KIE values indicate that these reactions proceed by similar mechanisms. We recently reported a KIE of 2.6 for an intermolecular ketone hydroacylation with non-chelating aldehydes, in which oxidative addition of Rh to the aldehyde C–H bond was rate limiting.^17^ Madsen reported a similar value of 2.85 for the oxidative addition of aldehydes to Rh(dppp)^+^.^18^ Lower KIE values of 1.4–1.7 are associated with hydroacylation reactions that involve rate-limiting migratory insertion or reductive elimination.^19,4e^ The large primary KIEs observed in the present case suggest that irreversible oxidative addition is likely the turnover-limiting step.

### D. Hammett studies

**Fig. 7:**
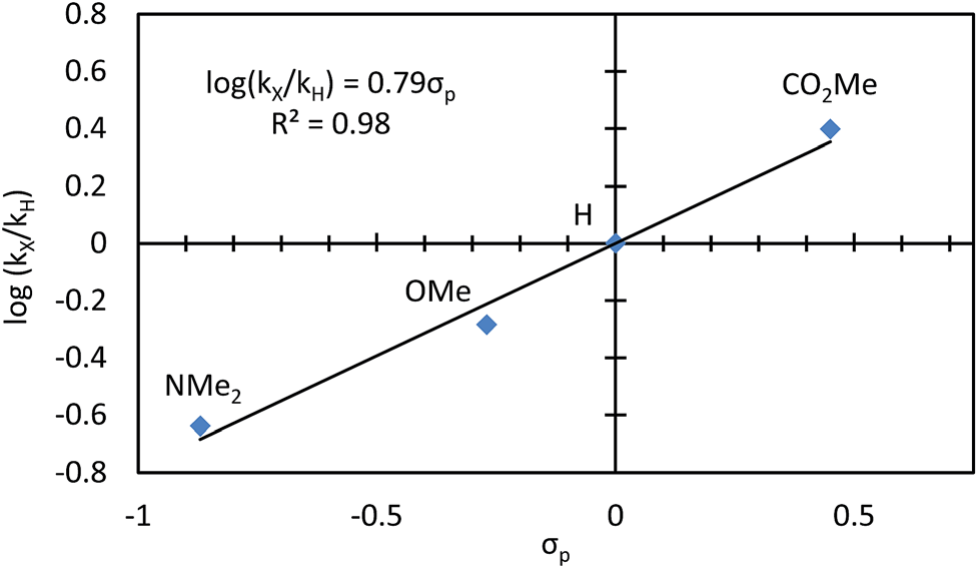
Hammett study for *para*-substituted benzaldehydes.

We conducted a Hammett study by reacting different *para*-substituted benzaldehydes with 4-nitro-2-vinylphenol (Fig. 7).^20,21^ Aldehydes with electron-withdrawing substituents reacted faster, while those with electron-donating groups reacted slower. A positive *ρ* value of 0.79 was measured, which indicates a build-up of negative charge in the transition state of the turnover-limiting step. This description is consistent with the transition state for oxidative addition of Rh to the aldehyde C–H bond.^22^

### E. Rationale for the ligand effects

Having established the catalyst resting states and the turnover-limiting step, we conclude that the rate of 2-vinylphenol hydroacylation is dictated predominantly by the rate of oxidative addition. Thus, the ligand dcpm likely accelerates this key step relative to its wide-bite-angle counterparts. To better understand the steric component of this effect,^23^ we examined the reactivity of the ligand (*t*Bu)_2_PCH_2_P(*t*Bu)(Me), which has a larger steric profile than dcpm but similar electronic properties and bite angle. This ligand did not provide an active catalyst. We thus propose that the small size of dcpm reduces steric repulsion in the transition state for oxidative addition. A pronounced electronic effect is also apparent, based on the comparison of dcpm with dppm (bis(diphenylphosphino)methane): faster rates were observed with the more basic disphosphine dcpm, despite its increased steric bulk. Our results contrast findings on the addition of H_2_ to Rh(diphosphine)_2_^+^ complexes, and the hydroacylation of alkenes with β-sulfur-substituted aldehydes. In the first case, wider bite angles favoured oxidative addition as a result of an electronic bite-angle effect;^24^ and in the latter case, small-bite-angle diphosphines promoted a rate-limiting reductive elimination.^4*e*^

### F. Proposed mechanism

**Fig. 8:**
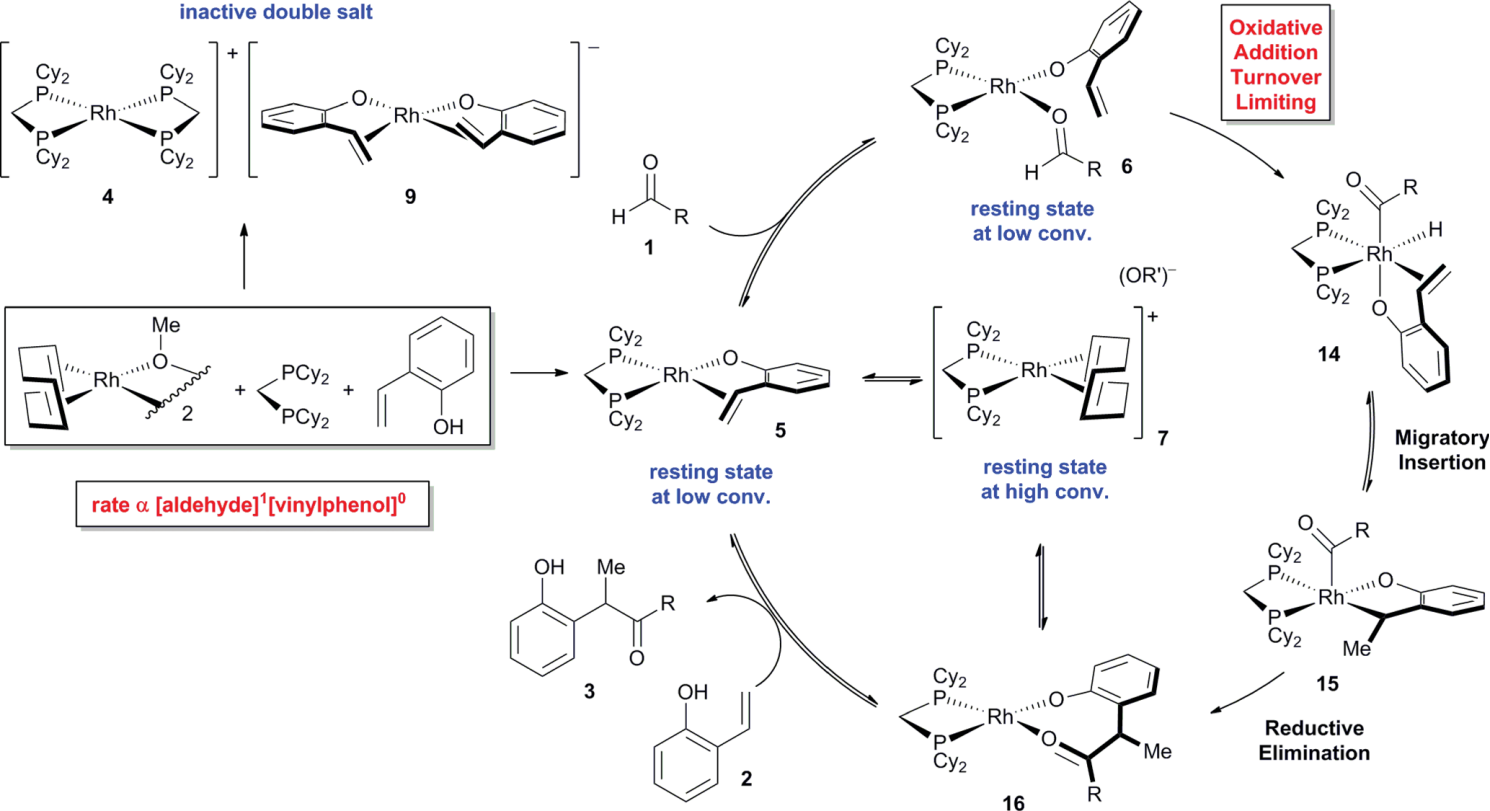
Proposed mechanism for vinylphenol hydroacylation (4-nitro group omitted from the vinylphenol, R = CH_2_Bn).

On the basis of our experimental evidence and literature precedence, we propose the mechanism shown in Fig. 8. The catalyst precursor [Rh(cod)OMe]_2_ reacts with dcpm and vinylphenol **2** to form either the catalytically inactive double salt of **4** and **9** or the major catalyst resting state **5**. Exchange of the olefin ligand for aldehyde **1** leads to intermediate **6**. Both complexes **5** and **6** are catalyst resting states and they decrease in concentration as the reaction progresses. Supported by kinetic isotope effects and a Hammett study, we propose that oxidative addition is turnover-limiting. The resulting acyl-Rh^III^-hydride **14** does not reductively decarbonylate to any measurable extent, but instead undergoes migratory insertion to yield intermediate **15**.^25^ Reductive elimination and displacement of the product **3** with substrate **2** completes the catalytic cycle. Alternatively, 1,5-cyclooctadiene can displace either the vinylphenol (**2**) or product (**3**) to form the off-cycle intermediate **7**, which increases in concentration during catalysis. The counterion of **7** is a dissociated phenolate anion that can hydrogen bond with excess substrate or product.

## Conclusions

We have disclosed a thorough mechanistic study on the branched-selective hydroacylation of 2-vinylphenols with a wide range of aryl, alkenyl, and alkyl aldehydes. Analysis of the catalyst resting states revealed that most of the catalyst is sequestered as an inactive double salt of Rh, while the active catalyst consists of a Rh(dcpm)(vinylphenolate) fragment. Non-directed oxidative addition of Rh to the aldehyde C–H bond occurs even at room temperature, followed by rapid hydroacylation. This strong binding of the vinylphenolate is likely a key to promoting hydroacylation over competitive aldehyde decarbonylation and aldol dehydration. KIE measurements and a Hammett study support oxidative addition as the turnover-limiting step. The high reactivity of neutral [Rh(X)(dcpm)] fragments likely arises from the electron-rich character of the complex, and small bite angle of the diphosphine, which promotes oxidative addition. In contrast, previous studies that use chelating aldehydes involve a fast and reversible C–H bond activation. This difference in mechanism highlights the challenge of non-directed aldehyde activation. Given the mild conditions of this transformation, [Rh(X)(dcpm)] fragments hold promise for future hydroacylations with non-chelating aldehydes.
