# Supplementary material for: Mechanistic insights into hydroacylation with non-chelating aldehydes
Source: Chem Sci. 2014 Sep 22;6(1):174–80. doi: 10.1039/c4sc02026j (PMC4285142; doi:10.1039/c4sc02026j)
Supplement: Supplementary file 1 [file SC-006-C4SC02026J-s001.pdf]

Electronic Supplementary Material (ESI) for Chemical Science.
This journal is © The Royal Society of Chemistry 2014

Supporting Information:

Mechanistic Insights into Hydroacylation with Non-chelating Aldehydes

Stephen K. Murphy[†,‡], Achim Bruch[†], Vy M. Dong*[,†]

<dongv@uci.edu>

[†]*Department of Chemistry, University of California, Irvine, California, 92697-2025, USA.*

[‡]*Department of Chemistry, University of Toronto, 80 St. George Street, Toronto, Ontario M5S 3H6, Canada.*

Table of Contents: **Page**

1.	General Considerations	S2
2.	Preparation of Substrates	S3
3.	Rh-catalyzed Hydroacylation to Synthesize Branched Ketones	S3
4.	Preparation of Organometallic Complexes	S4
5.	NMR spectra	S7
6.	Crystallographic Data	S22

1 General Considerations

All reactions were run in oven-dried or flame-dried glassware under an atmosphere of N_2. Solvents used in hydroacylations were purchased as anhydrous grade, degassed by three freeze-pump-thaw cycles, and stored over 3 Å MS within an N_2 filled glove box. Solvents used for substrate synthesis were purified using an Innovative Technologies Pure Solv system. All of the vinylphenols used in this study were described in our previous report (Murphy, S.K.; Bruch, A.; Dong, V.M. *Angew. Chem. Int. Ed.* **2014**, *53*, 2455.). The molarity of organolithium reagents was determined by titration with *iso*-propanol/1,10-phenanthroline. Liquid aldehydes were distilled under reduced pressure and degassed by three freeze-pump-thaw cycles prior to use. Solid aldehydes were purified either by washing with saturated aqueous sodium bicarbonate solution or by column chromatography. Reactions were monitored either via gas chromatography-mass spectrometry (GC-MS) using an Agilent Technologies 7890A GC system equipped with an Agilent Technologies 5975C inert XL EI/CI MSD, or by analytical thin-layer chromatography on EMD Silica Gel 60 F_{254} plates. Visualization of the developed plates was performed under UV light (254 nm) or $KMnO_4$ stain. Column chromatography was performed with Silicycle Silia-P Flash Silica Gel using glass columns. Automated column chromatography was performed using either a Biotage SP1 or Teledyne Isco CombiFlash Rf 200 purification system. ^{1}H, ^{13}C, ^{19}F, and ^{31}P NMR spectra were recorded on a Bruker DRX-400 (400 MHz ^{1}H, 100 MHz ^{13}C, 376.5 MHz ^{19}F, 161.9 ^{31}P), GN-500 (500 MHz ^{1}H, 125.7 MHz ^{13}C) or CRYO-500 (500 MHz ^{1}H, 125.7 MHz ^{13}C) spectrometer. ^{1}H NMR spectra were internally referenced to the residual solvent signal or TMS. ^{13}C NMR spectra were internally referenced to the residual solvent signal. Data for ^{1}H NMR are reported as follows: chemical shift (δ ppm), multiplicity (s = singlet, d = doublet, t = triplet, q = quartet, m = multiplet, br = broad), coupling constant (Hz), integration. Data for ^{13}C NMR are reported in terms of chemical shift (δ ppm). Infrared spectra were obtained on a Thermo Scientific Nicolet iS5 FT-IR spectrometer equipped with an iD5 ATR accessory. High resolution mass spectra (HRMS) was performed by the University of California, Irvine Mass Spectrometry Center. X-ray crystallography was performed by the University of California, X-ray Crystallography Facility.

2 Preparation of substrates

d_1-2-naphthaldehyde

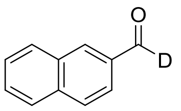

To a round bottom flask equipped with a stir bar was added sequentially N-(methoxy)methylammonium chloride (0.512 g, 5.25 mmol, 1 equiv), DMAP (0.064 g, 0.525 mmol, 0.1 equiv), dichloromethane (20 mL), triethylamine (1.536 mL, 11.02 mmol, 2.1 equiv), and 2-naphthoyl chloride (1.000 g, 5.25 mmol, 1 equiv). The solution was stirred for 10 minutes and then concentrated under reduced pressure. Ethyl acetate was added and the solution was washed twice with saturated NH_4Cl(aq) and then $NaHCO_3$(aq) solution. The organic layer was dried with $MgSO_4$ and then filtered and concentrated under reduced pressure to give the weinreb amide as a clear light yellow oil (0.920 g, 81 % yield) which was used without further purification. The weinreb amide was added to a second round bottom flask equipped with a stir bar and septum, along with THF (25 mL). The flask was lowered into an ice bath, and then $LiAlD_4$ (0.197 g, 4.70 mmol, 1.1 equiv) was added slowly. After 10 minutes, the reaction was worked up using the Feiser and Feiser method and purified by column chromatography to give the product as a white solid (0.636 g, 95%). ^{1}H NMR data matched those previously reported (Fujihara, T.; Cong, C.; Iwai, T.; Terao, J.; Tsuji, Y. *Synlett* **2012**, *23*, 2389). **^{1}H NMR** (500 MHz, $CDCl_3$) δ 8.35 (s, 1H), 8.02 (d, *J* = 8.1 Hz, 1H), 8.00 – 7.87 (m, 3H), 7.66 (t, *J* = 7.4 Hz, 1H), 7.60 (t, *J* = 7.1 Hz, 1H).

***d_1*-hydrocinnamaldehyde**

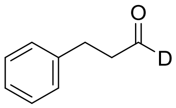

The title compound was synthesized according to a literature procedure (Jean, M.; Renault, J.; Uriac, P.; Capet, M.; van de Weghe, P. *Org. Lett.* **2007**, *9*, 3623). **^{1}H NMR** (400 MHz, $CDCl_3$) δ 7.39 – 7.10 (m, 5H), 2.96 (t, *J* = 7.6 Hz, 2H), 2.78 (t, *J* = 7.6 Hz, 2H).

3 Rh-catalyzed Hydroacylation to Synthesize Branched Ketones

The hydroxyketone products of this study can exist in equilibrium with two diastereomeric hemiketal forms in widely varying ratios and this is noted where appropriate in the characterization data. This phenomenon can give rise to very complicated NMR spectra because purified compounds can exist in three different isomeric forms. For more examples of this phenomenon, see (Murphy, S.K.; Bruch, A.; Dong, V.M. *Angew. Chem. Int. Ed.* **2014**, *53*, 2455.).

4-(2-hydroxy-5-nitrophenyl)-1-phenylpentan-3-one-5-*d*

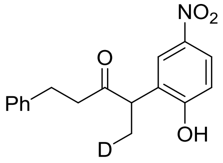

To a 1 dram vial was added 4 mol % $[Rh(COD)OMe]_2$ and 8 mol % dcpm. The vinyl phenol (0.2 mmol, 1 equiv), aldehyde (1.5 equiv), and THF (200 μL) were added to the vial which was then sealed with a Teflon-lined screw cap. The reaction was heated to 60 °C for 24 hours and then cooled to room temperature. The branched to linear ratio was determined by integration of the methine proton (quartet) of the branched product versus the methylene protons of the linear product (triplets) in the crude ^{1}H NMR spectrum. The product was isolated by preparatory TLC as a colorless oil (54.0 mg, 90%). This compound has very concentration dependent ^{1}H NMR spectrum and sharp peaks can only be obtained under very dilute conditions. The additional peaks in the

^{1}H NMR correspond to hemiketal isomers of the product. Only the peaks corresponding to the open-chain form are tabulated here. **^{1}H NMR** (400 MHz, $CDCl_3$) δ 8.08 (d, J = 8.9 Hz, 1H), 7.97 (s, 1H), 7.30 – 7.23 (m, 2H), 7.22 – 7.09 (m, 3H), 6.92 (d, J = 8.9 Hz, 1H), 3.92 (br s, 1H), 3.10 – 2.69 (m, 4H), 1.46 (d, J = 7.3 Hz, 2H). **^{2}H NMR** (77 MHz, $CDCl_3$) δ 1.47. **^{13}C NMR** (126 MHz, $CDCl_3$) δ 213.96, 160.51, 141.14, 140.21, 128.50, 128.18, 126.59, 126.24, 125.38, 124.98, 117.03, 48.51, 43.06, 29.52, 15.20. **LRMS** (ESI): calcd for $[C_{17}H_{15}DNO_4]^-$ 299.11 (100%), 300.12 (18.4%), 301.12 (1.6%), found 298.0 (2.0%), 299.3 (100%), 300.31 (17.0%), 301.35 (2.0%).

4-(2-hydroxy-5-nitrophenyl)-1-phenylpentan-3-one-5-*d*

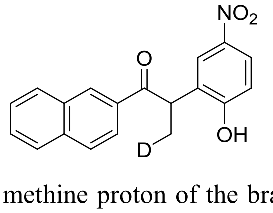

To a 1 dram vial was added 4 mol % $[Rh(COD)OMe]_2$ and 8 mol % dcpm. The vinyl phenol (0.2 mmol, 1 equiv), aldehyde (1.5 equiv), and 1,4-dioxane (200 µL) were added to the vial which was then sealed with a Teflon-lined screw cap. The reaction was heated to 100 °C for 24 hours and then cooled to room temperature. The branched to linear ratio was determined by integration of the methine proton of the branched product versus the methylene protons of the linear product in the crude ^{1}H NMR spectrum. The product was isolated by preparatory TLC as a white solid (50.3 mg,78%). The compound was isolated with a 92:8 branched:linear ratio. **^{1}H NMR** (500 MHz, $CDCl_3$) *Linear isomer*: δ 9.36 (s, 1H), 8.71 (s, 1H), 8.25 (d, J = 1.8 Hz, 1H), 8.18 – 7.87 (m, 4H), 7.67 (t, J = 7.4 Hz, 1H), 7.61 (t, J = 7.4 Hz, 1H), 7.03 (d, J = 8.9 Hz, 1H), 5.28 (t, J = 6.9 Hz, 1H), 1.72 (d, J = 6.9 Hz, 1H). *Branched isomer*: δ 3.69 (d, J = 6.0 Hz, 1H), 3.17 (t, J = 6.0 Hz, 1H). **^{2}H NMR** (77 MHz, $CDCl_3$) δ 1.70. **^{13}C NMR** (126 MHz, $CDCl_3$) δ 204.55, 161.05, 141.25, 136.17, 132.46, 132.43, 131.36, 129.97, 129.43, 129.07, 127.88, 127.25, 126.23, 125.18, 124.14, 117.91, 44.23, 17.21 (t, J = 20.2 Hz).

4 Preparation of Organometallic Complexes

$[Rh(dcpm)]_2Cl$

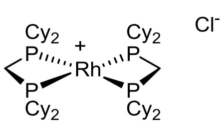

To a 1 dram vial was added $[Rh(COD)Cl]_2$ (20 mg, 0.041 mmol, 1 equiv) and dcpm (66 mg, 0.162 mmol, 4 equiv). Chloroform (0.5 mL) was added and a homogenous solution was obtained. Precipitate began to form after approximately 30 seconds. Hexanes was added and the product was collected by decantation. The powder was triturated with hexanes and decanted twice. The product was obtained as an orange powder (33.6 mg, 86 % yield). **^{1}H NMR** (400 MHz, $CDCl_3$) δ 3.08 – 3.00 (m, 4H), 2.28 – 1.05 (m, 88H). **^{31}P NMR** (162 MHz, $CDCl_3$) δ -16.9 (d, J = 110.8 Hz). **LRMS** (ESI) calcd for $[C_{50}H_{92}P_4Rh]$+ 919.5, found 919.5.

$[Rh(4\text{-nitro-2-vinyl-phenolate})_2][(18\text{-crown-6})K]$

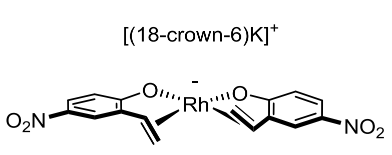

To a 1 dram vial was added $[Rh(COD)OMe]_2$ (10 mg, 0.0207 mmol, 1 equiv), 4-nitro-2-vinylphenol (13.6 mg, 0.0826 mmol, 4 equiv), ^{t}BuOK (4.6 mg, 0.0414 mmol, 2 equiv), and 18-crown-6 (10.9 mg, 0.0414 mmol, 2 equiv). THF (0.650 mL) was added and the solution was shaken for 5 minutes. Hexanes was added to afford a yellow solid which was decanted, triturated with hexanes, decanted again, and finally dried under vacuum. The product was

obtained as a yellow solid (21 mg, 67% yield). X-ray quality crystals were obtained after two rounds of crystallization from saturated THF/Hexanes solutions. **^{1}H NMR** (600 MHz, d_8-THF) δ 7.78 (dd, J = 9.0, 2.8 Hz, 2H), 7.67 (d, J = 2.8 Hz, 2H), 6.19 (d, J = 9.0 Hz, 2H), 3.65 (dd, J = 12.1, 8.0 Hz, 2H), 3.61 (s, 24H), 3.50 (dd, J = 12.1, 2.6 Hz, 2H), 2.08 (d, J = 8.0 Hz, 2H). **^{13}C NMR** (126 MHz, d_8-THF) δ 180.10, 136.31, 135.73, 126.11, 123.06, 116.42, 75.34 (d, J = 12.0 Hz), 71.27, 58.06 (d, J = 15.4 Hz). **LRMS** (ESI) calcd for $[C_{16}H_{12}N_2O_6Rh]^-$ 431.0, found 431.0.

$[Rh(dcpm)]_2[Rh(4\text{-nitro-2-vinyl-phenolate})_2]$

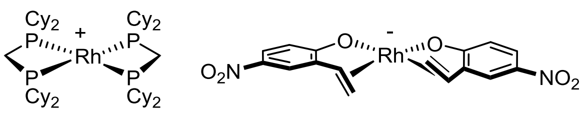

To a 1 dram vial was added $[Rh(COD)OMe]_2$ (10 mg, 0.0207 mmol, 1 equiv), dcpm (16.9 mg, 0.0413 mmol, 2 equiv), and 4-nitro-2-vinylphenol (6.8 mg, 0.0413 mmol, 2 equiv). THF (0.400 mL) was added and the solution was heated to 60 °C until the reaction became homogeneous. The solution was cooled to room temperature and filtered into a separate vial. Hexanes (2 mL) was added to the solution and then the vial was sealed and placed in a -30 °C freezer for 24 hours. The precipitate was decanted, triturated with hexanes, decanted again, and finally dried under reduced pressure. The product was obtained as an orange powder (35.0 mg, 75 % yield) contaminated with approximately 10% of $[Rh(dcpm)_2]^+$(4-nitro-2-vinylphenolate)$^-$. Spectroscopic data were very similar to $[Rh(dcpm)]_2Cl$ and $[Rh(4\text{-nitro-2-vinyl-phenolate})_2]$[(18-crown-6)K]. **^{1}H NMR** (600 MHz, d_8-THF) δ 7.77 (dd, J = 9.1, 2.8 Hz, 2H), 7.66 (d, J = 2.8 Hz, 2H), 6.13 (d, J = 9.1 Hz, 2H), 3.64 – 3.56 (m, 2H), 3.49 (dd, J = 12.1, 2.2 Hz, 1H), 3.26 – 3.20 (m, 4H), 2.19 (d, J = 11.8 Hz, 8H), 2.05 (d, J = 7.9 Hz, 2H), 1.97 – 1.20 (m, 80H). **^{13}C NMR** (126 MHz, d_8-THF) δ 181.30, 136.69, 136.47, 126.91, 123.72, 116.88, 75.92 (d, J = 11.9 Hz), 58.52 (d, J = 15.2 Hz), 38.41 (d, J = 5.1 Hz), 31.46, 30.10, 29.23 – 28.97 (m), 28.76 – 28.63 (m), 27.76 (s). **^{31}P NMR** (162 MHz, d_8-THF) δ -17.2 (d, J = 110.7 Hz). **LRMS** (ESI) calcd for $[C_{50}H_{92}P_4Rh]^+$ 919.5, found 919.5; calcd for $[C_{16}H_{12}N_2O_6Rh]^-$ 431.0, found 431.0.

$[Rh(dcpm)(COD)]BF_4$

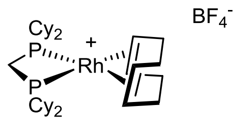

A solution of dcpm (20 mg, 0.0493 mmol, 1 equiv) in DCM (1.5 mL) was added dropwise to a solution of $[Rh(COD)_2]BF_4$ (20 mg, 0.0493 mmol, 1 equiv) in DCM (1.5 mL) in a 20 mL vial. The resulting solution was filtered and the solvent was removed under reduced pressure. The solid was triturated with hexanes and decanted. The solid was dissolved in chloroform and THF and then precipitated with hexanes. The product was isolated as an orange powder (25 mg, 69 % yield). **^{1}H NMR** (400 MHz, $CDCl_3$) δ 5.37 (s, 4H), 3.28 (t, J = 9.9 Hz, 2H), 2.33 (s, 8H), 2.18 – 1.13 (m, 44H). **^{31}P NMR** (162 MHz, $CDCl_3$) δ -27.1 (d, J = 125.0 Hz). **LRMS** (ESI) calcd for $[C_{33}H_{58}P_2Rh]^+$ 619.3, found 619.2.

[Rh(dcpm)(4-nitro-2-vinylphenolate)]

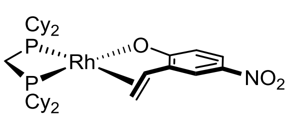

To a 1 dram vial was added $[Rh(dcpm)(COD)]BF_4$ (6.4 mg, 0.0089 mmol, 1 equiv.), 4-nitro-2-vinylphenol (1.5 mg, 0.0089 mmol, 1 equiv.) and ^{t}BuOK (1.0 mg, 0.0089 mmol). d_8-THF (0.65 mL) was added and a white precipitate formed (KBF_4). The solution was transferred to an NMR tube and sealed with a septum and parafilm. The product was formed in ca. 98% NMR yield. Repeating this experiment on larger scale lead to NMR yields that varied from 90-99%. Attempts to crystallize this compound were unsuccessful. The

coupling of the carbons of the olefin and both Rh and P in the ^{13}C NMR are characteristic of the title compound. **^{1}H NMR** (400 MHz, d_8-THF) δ 7.69 (d, *J* = 9.2 Hz, 1H), 7.61 (s, 1H), 6.08 (d, *J* = 9.2 Hz, 1H), 5.49 – 5.40 (m, 1H), 4.63 (d, *J* = 14.8 Hz, 1H), 4.51 – 4.42 (m, 1H), 3.02 – 2.72 (m, *J* = 27.5 Hz, 2H), 2.44 – 0.71 (m, 44H). **^{13}C NMR** δ 135.44, 132.83, 130.08, 127.17, 124.62, 118.03, 94.94 – 94.64 (m), 78.49 – 78.10 (m), 32.60, 31.7 – 31.3 (m), 29.72, 28.92 (d, *J* = 12.0 Hz), 27.84. **^{31}P NMR** (162 MHz, $CDCl_3$) δ 0.41 (dd, *J* = 125.4, 92.3 Hz), -27.15 (dd, *J* = 147.9, 92.3 Hz).

[Rh(dcpm)(4-nitro-2-vinylphenolate)(hydrocinnamaldehyde)]

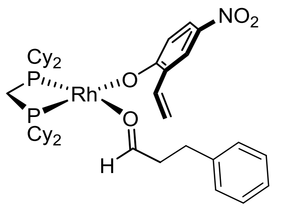

To the NMR sample from the synthesis of [Rh(dcpm)(4-nitro-2-vinylphenolate)] (above) was added 5 µL of hydrocinnamaldehyde. The title compound could be observed in small quantities (ca. 10 %) transiently before being converted to the hydroacylation product below. Larger quantities could be formed if excess 4-nitro-2vinylphenol and aldehyde were added to establish a steady state concentration of the title compound. However, due to the high concentration of reagents required to form this complex, we could only characterize it by ^{31}P NMR. We assign the structure as the title compound by comparison of the chemical shift and coupling constants to [Rh(dcpm)(4-nitro-2-vinylphenolate)]. Both the title compound and [Rh(dcpm)(4-nitro-2-vinylphenolate)] display a ^{31}P peak at about 1 ppm with a coupling constant to Rh of ca. 125 Hz. We assign this peak as the phosphine trans to the phenoxide. A new peak at -9.9 ppm is present in the title compound which we assign as the phosphine trans to the aldehyde. **^{31}P NMR** (162 MHz, $CDCl_3$) δ 1.7 (dd, *J* = 126, 69 Hz), -9.9 (dd, *J* = 117, 69 Hz).

[Rh(dcpm)(COD)][4-nitro-2-(3-oxo-5-phenlypentan-2-yl)phenolate]

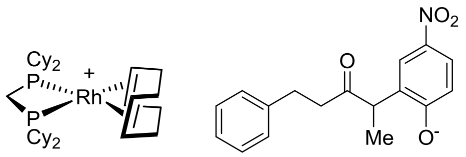

The title compound was synthesized according to the same procedure for [Rh(dcpm)(4-nitro-2-vinylphenolate)(hydrocinnamaldehyde)] except that a large excess of hydrocinnamaldehyde (100 µL) was added which resulted in immediate hydroacylation at room temperature (ca. 80% yield). ^{31}P analysis showed a doublet at -27.1 ppm indicative of the $[Rh(dcpm)(COD)]^+$ fragment based on analogy to $[Rh(dcpm)(COD)]BF_4$. Analysis of the reaction mixture by ESI MS confirmed formation of $[Rh(dcpm)(COD)]^+$ and the anion of the hydroacylation product. As well, a dimer of the hydroacylation product and its phenolate was observed by ESI MS when both excess aldehyde and olefin were added. **^{31}P NMR** (162 MHz, $CDCl_3$) δ -26.95 (d, *J* = 125.7 Hz). **LRMS** (ESI) calcd for $[C_{33}H_{58}P_2Rh]+$ 619.3, found 619.2. Calcd for $[C_{17}H_{16}NO_4]-$ 298.1, found 298.3. Calcd for $[C_{34}H_{33}N_2O_8]-$ 597.2, found 597.4.

8 NMR Spectra

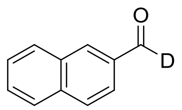

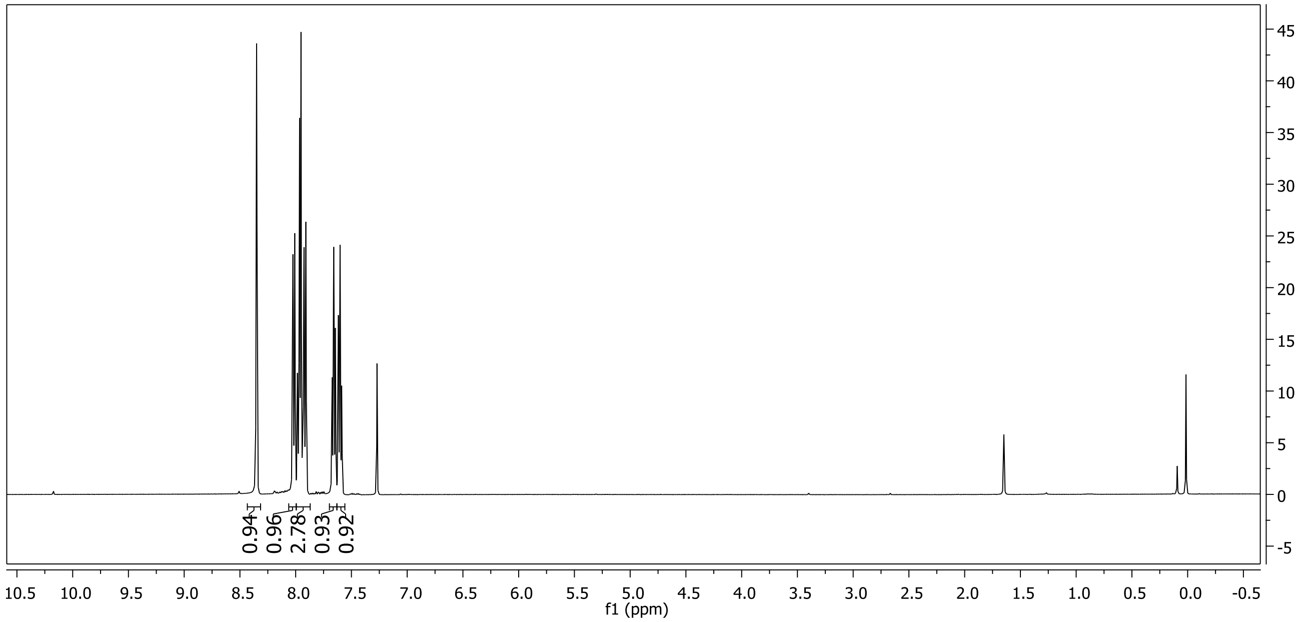

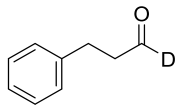

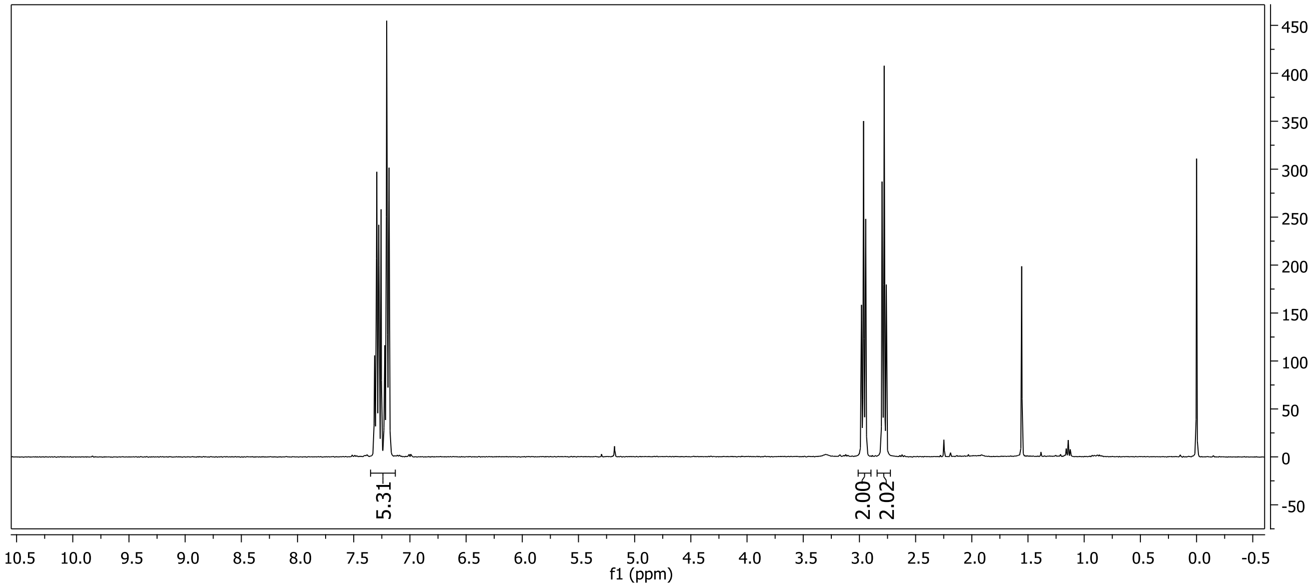

5.31
2.00
2.02
450
400
350
300
250
200
150
100
50
0
-50
10.5
10.0
9.5
9.0
8.5
8.0
7.5
7.0
6.5
6.0
5.5
5.0
4.5
4.0
3.5
3.0
2.5
2.0
1.5
1.0
0.5
0.0
-0.5
f1 (ppm)

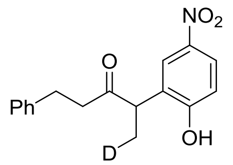

NO2
O
Ph
D
OH

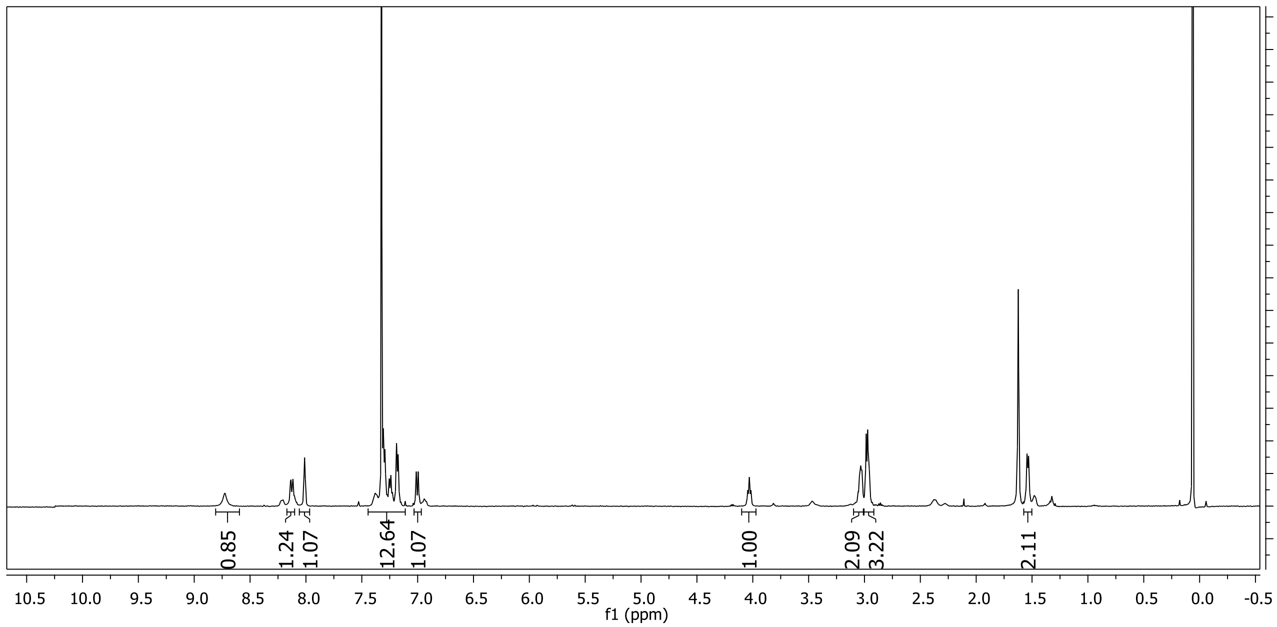

0.85
1.24
1.07
12.64
1.07
1.00
2.09
3.22
2.11
f1 (ppm)

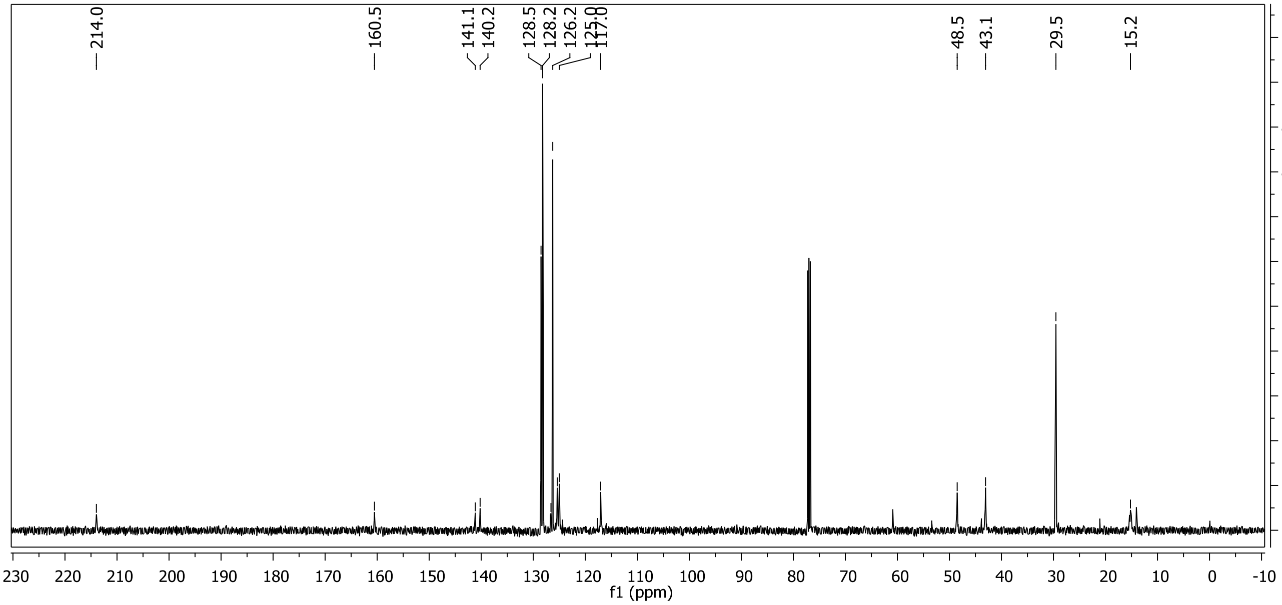

214.0
160.5
141.1
140.2
128.5
128.2
126.2
125.0
117.0
48.5
43.1
29.5
15.2
f1 (ppm)

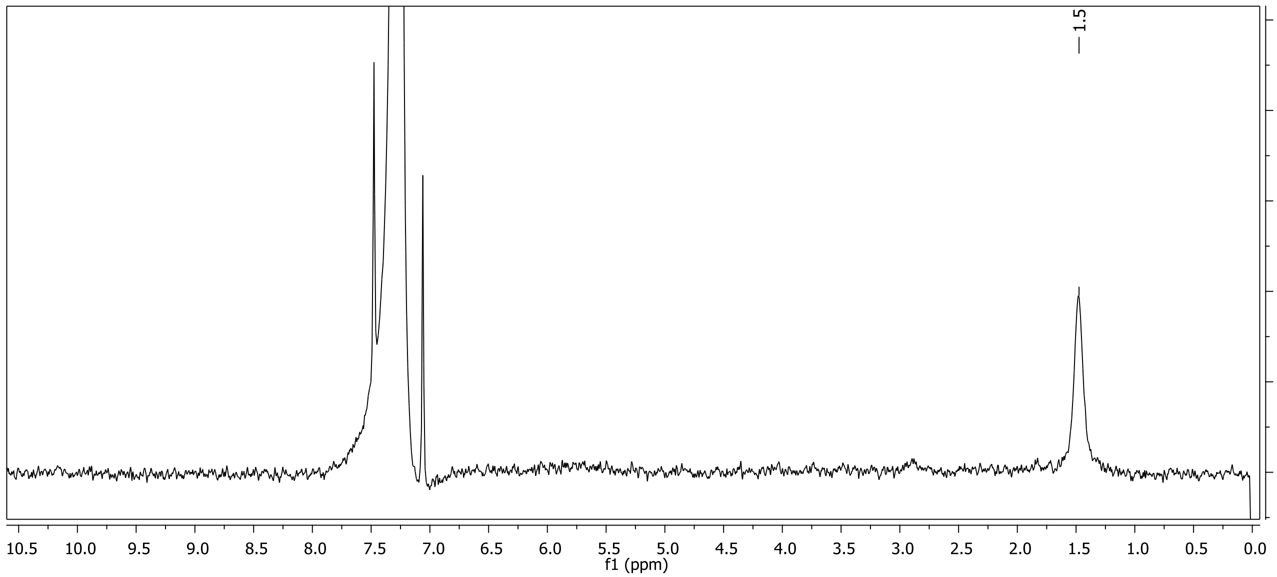

1.5
f1 (ppm)

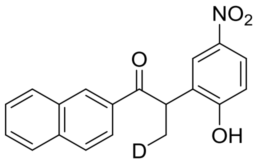
NO2
O
D
OH

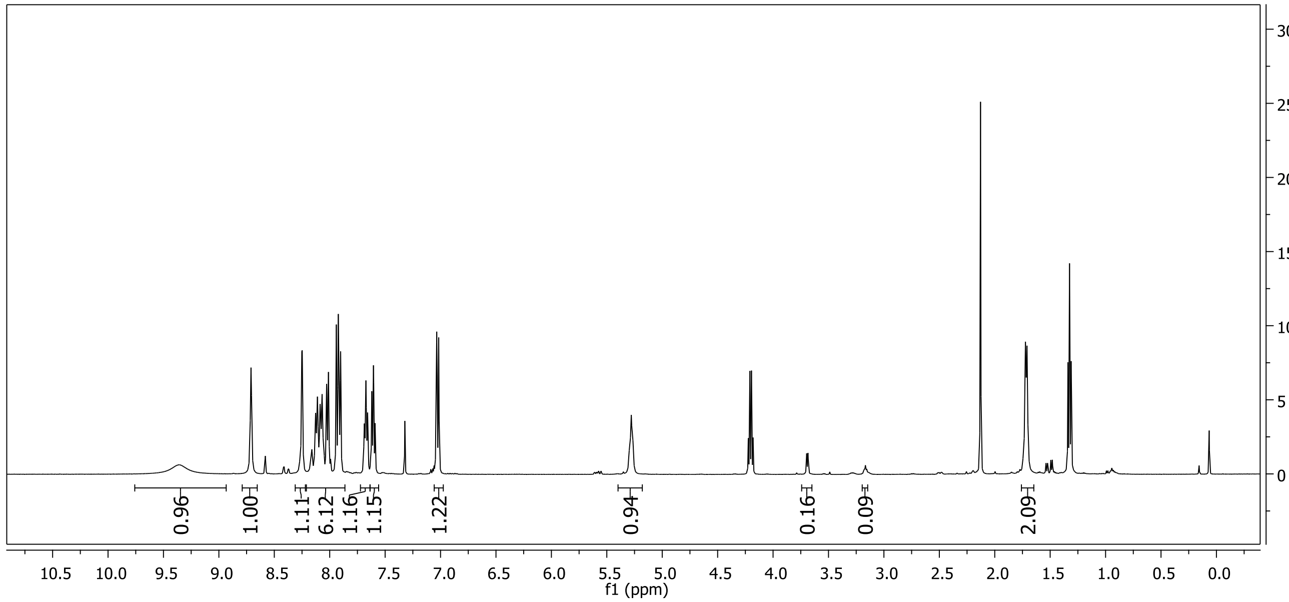
0.96
1.00
1.11
6.12
1.16
1.15
1.22
0.94
0.16
0.09
2.09
f1 (ppm)

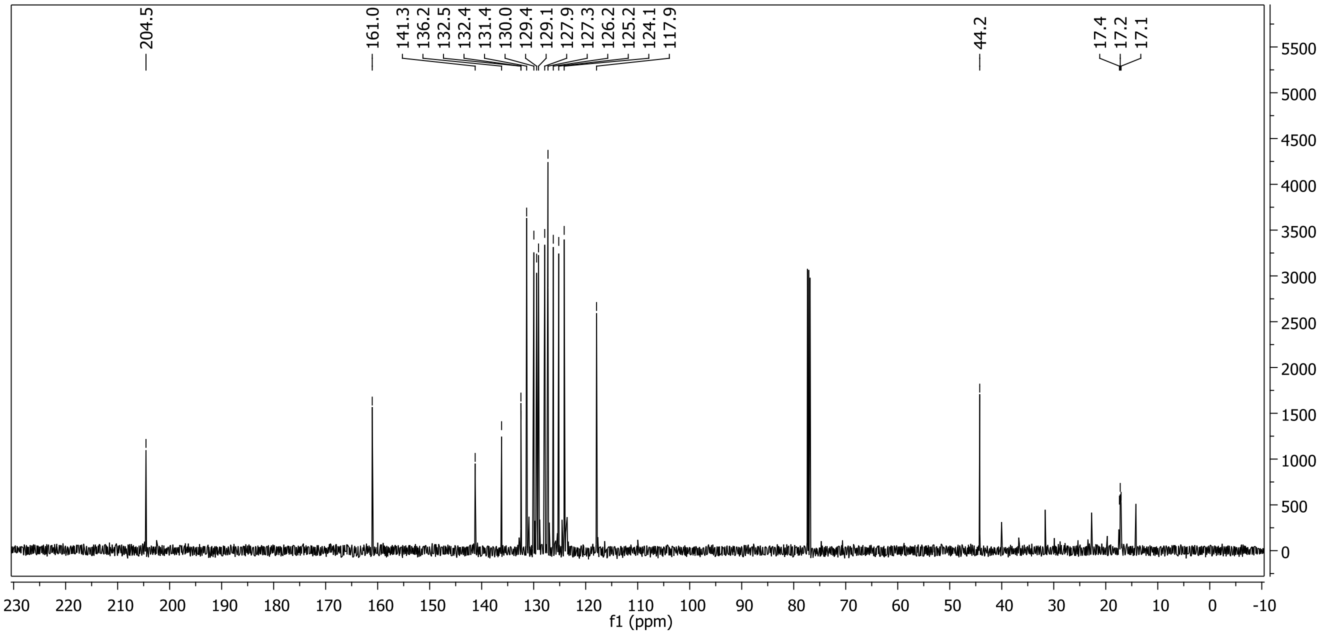
204.5
161.0
141.3
136.2
132.5
132.4
131.4
130.0
129.4
129.1
127.9
127.3
126.2
125.2
124.1
117.9
44.2
17.4
17.2
17.1
f1 (ppm)

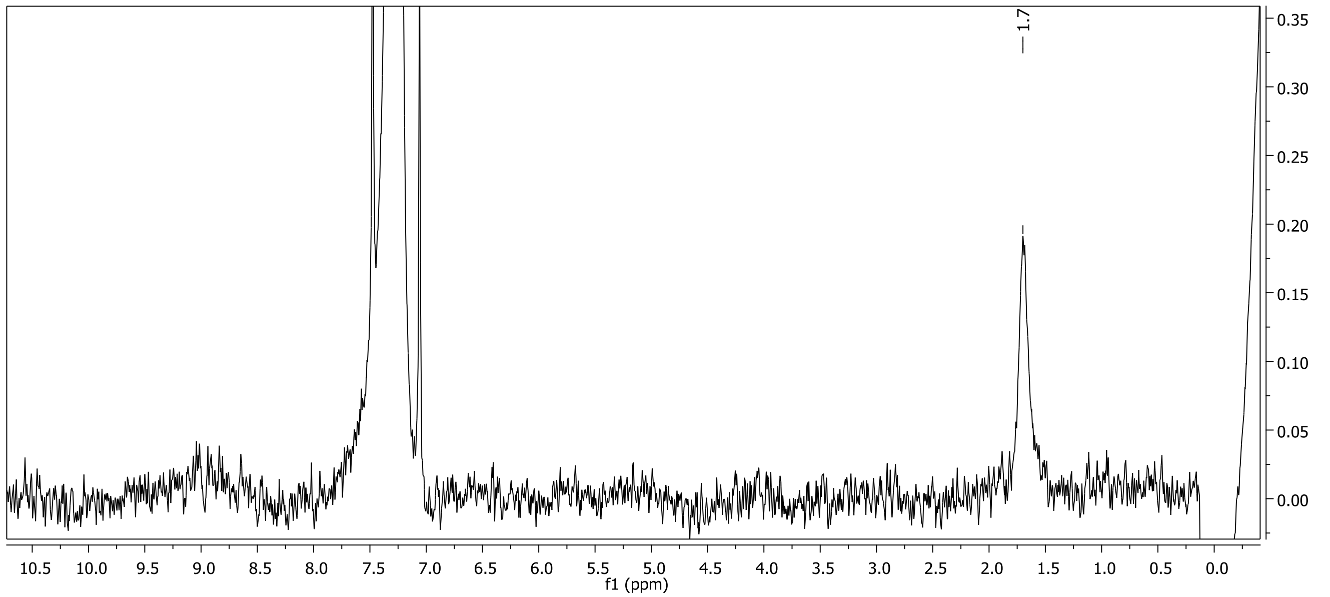

1.7
0.35
0.30
0.25
0.20
0.15
0.10
0.05
0.00
10.5 10.0 9.5 9.0 8.5 8.0 7.5 7.0 6.5 6.0 5.5 5.0 4.5 4.0 3.5 3.0 2.5 2.0 1.5 1.0 0.5 0.0
f1 (ppm)

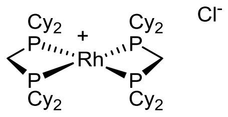

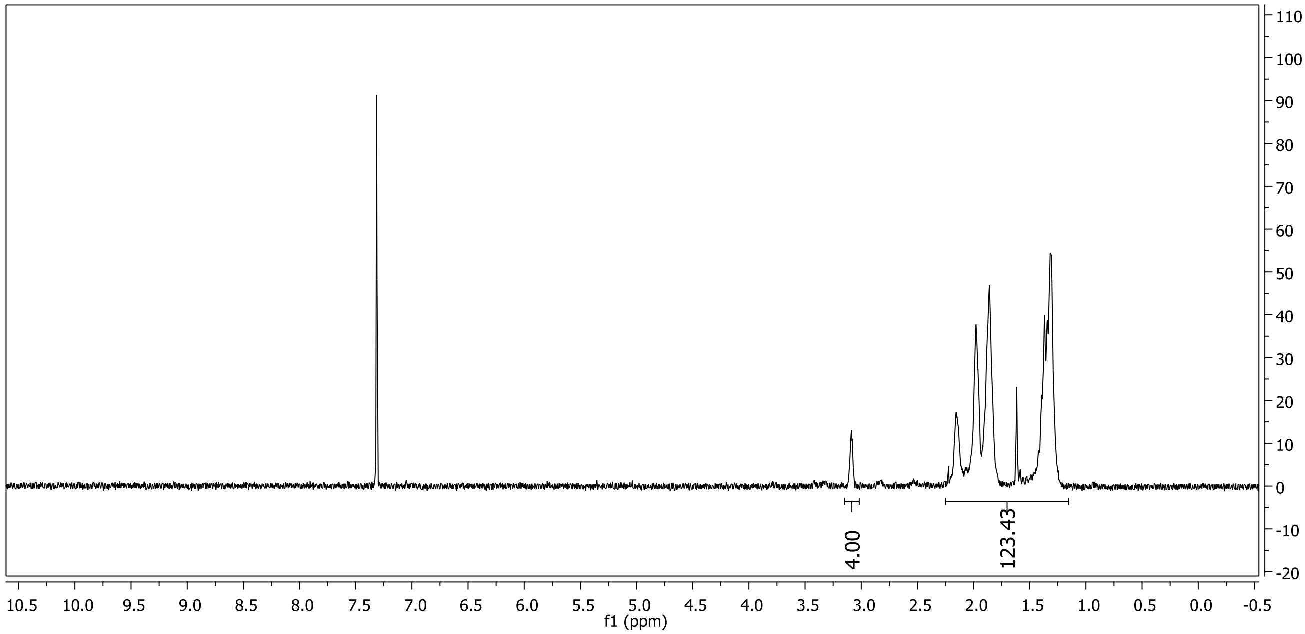

4.00
123.43
f1 (ppm)

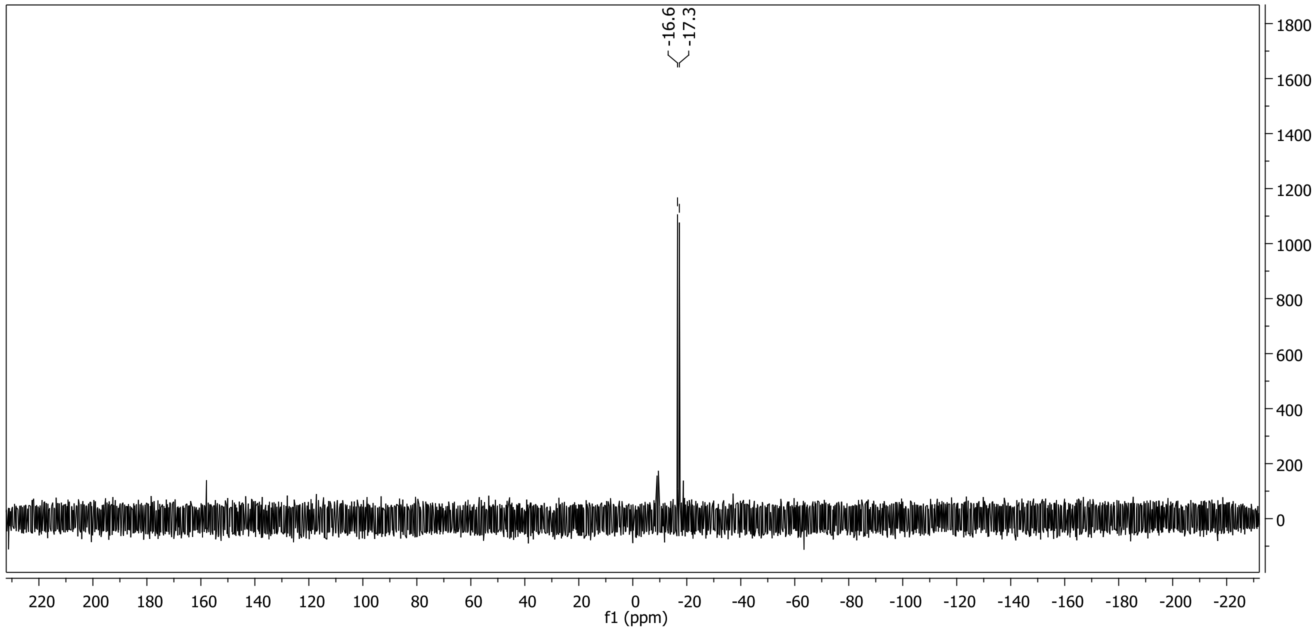

-16.6
-17.3
f1 (ppm)

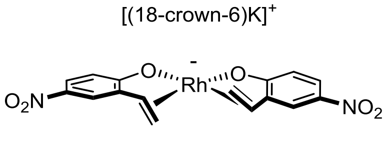
[(18-crown-6)K]+
O
Rh
O
O_2N
NO_2

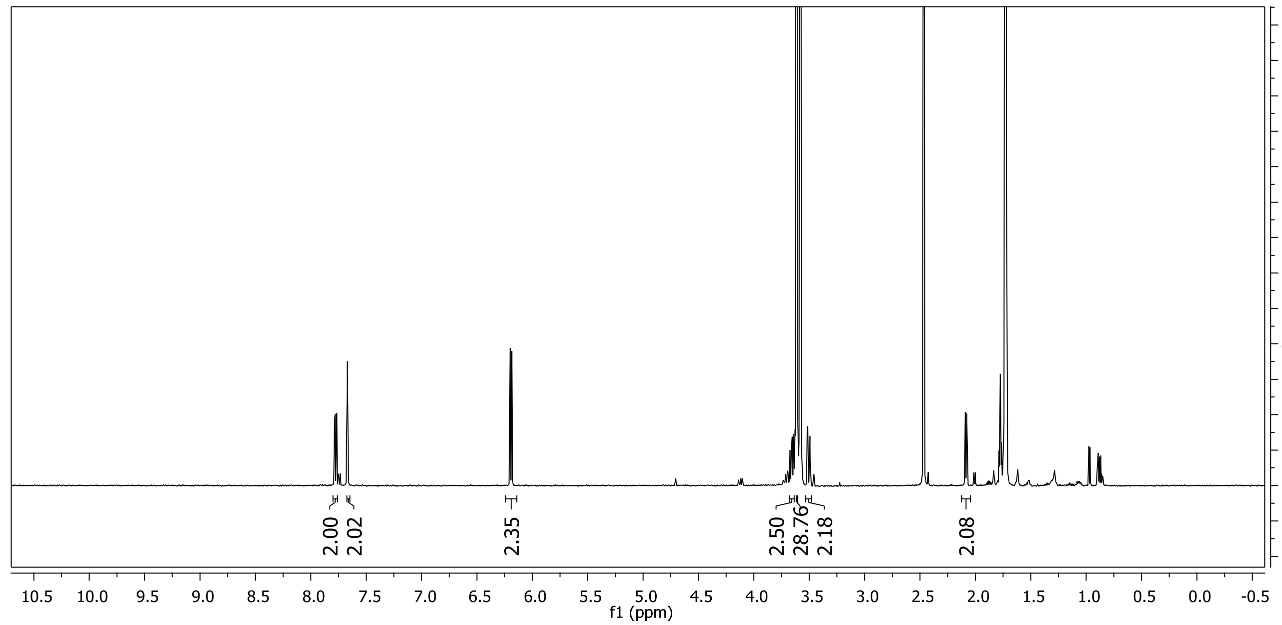
2.00
2.02
2.35
2.50
28.76
2.18
2.08
f1 (ppm)

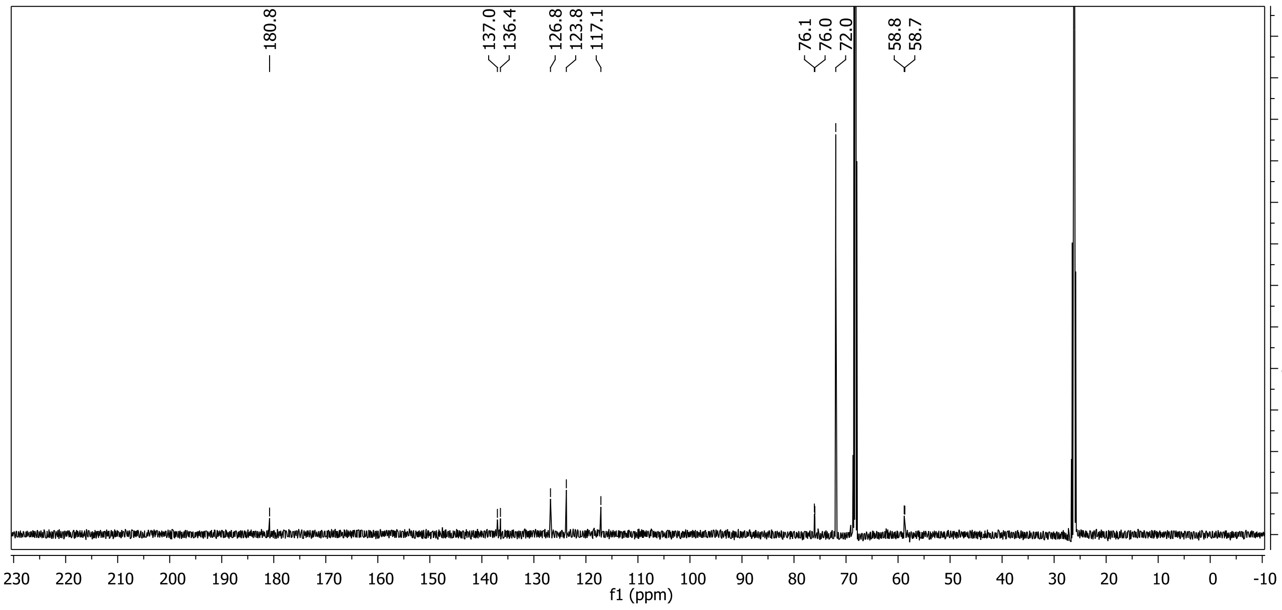
180.8
137.0
136.4
126.8
123.8
117.1
76.1
76.0
72.0
58.8
58.7
f1 (ppm)

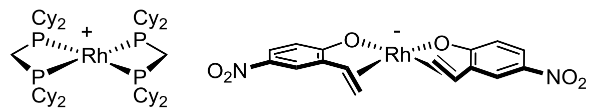
Cy2
P
Rh
P
Cy2
+
Cy2
P
P
Cy2
O2N
O
-
Rh
O
NO2

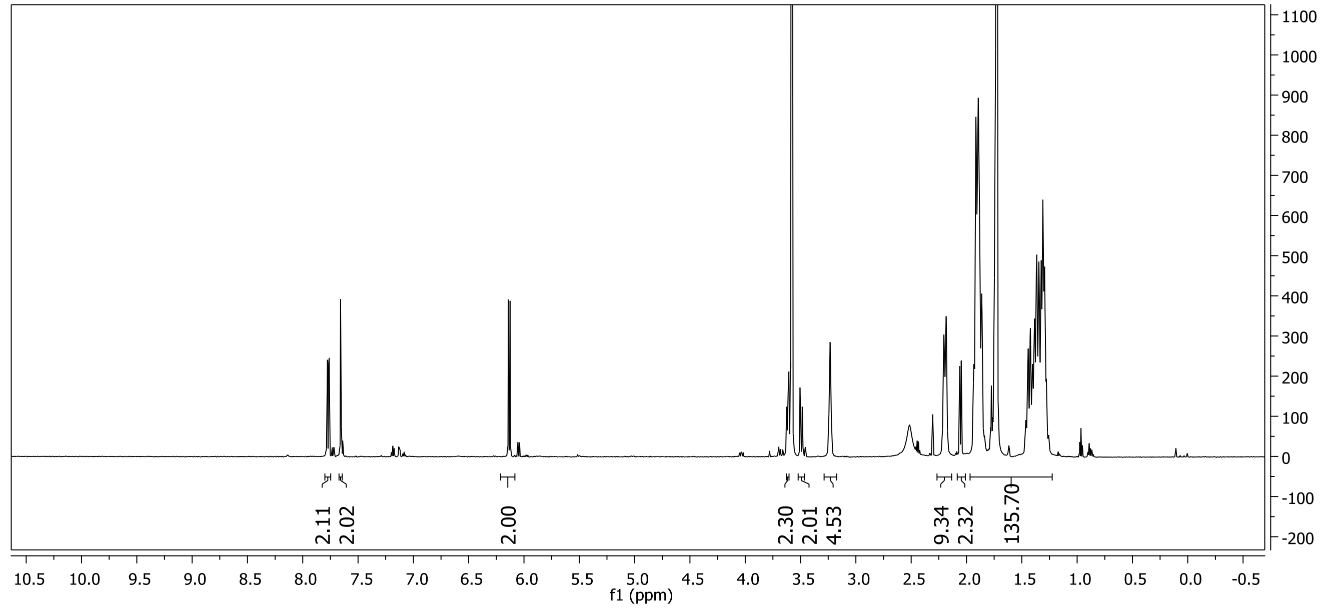
2.11
2.02
2.00
2.30
2.01
4.53
9.34
2.32
135.70
f1 (ppm)

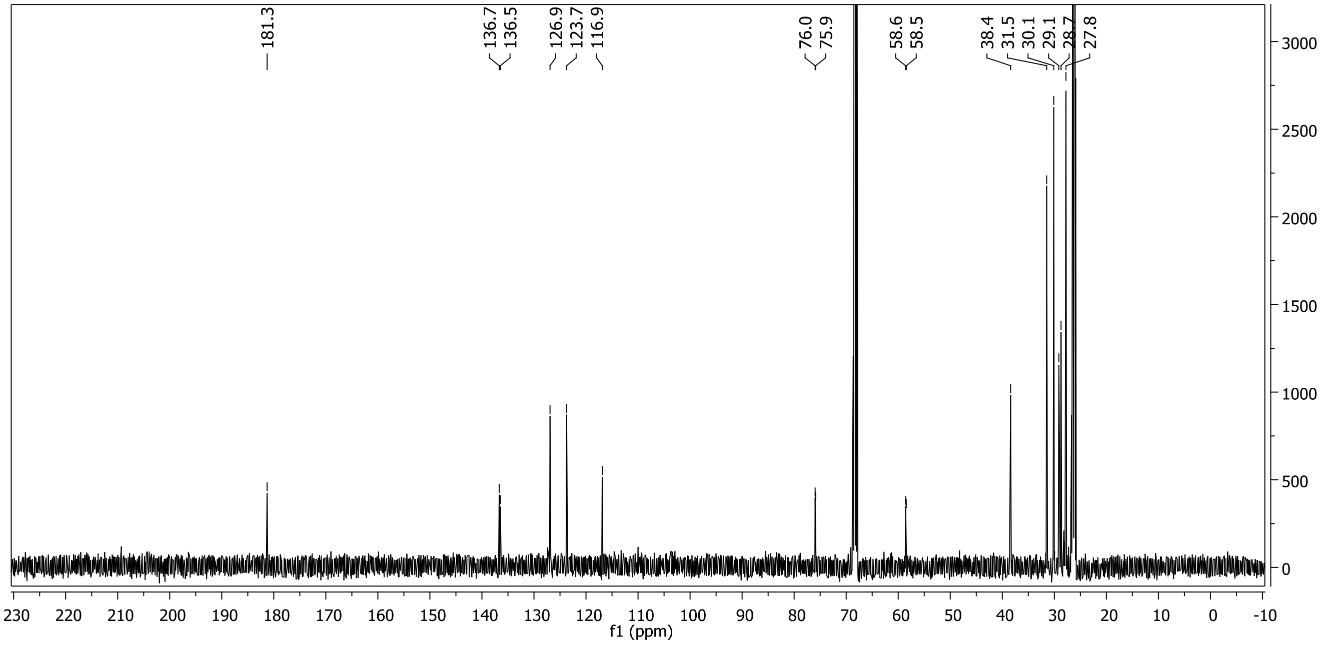
181.3
136.7
136.5
126.9
123.7
116.9
76.0
75.9
58.6
58.5
38.4
31.5
30.1
29.1
28.7
27.8
f1 (ppm)

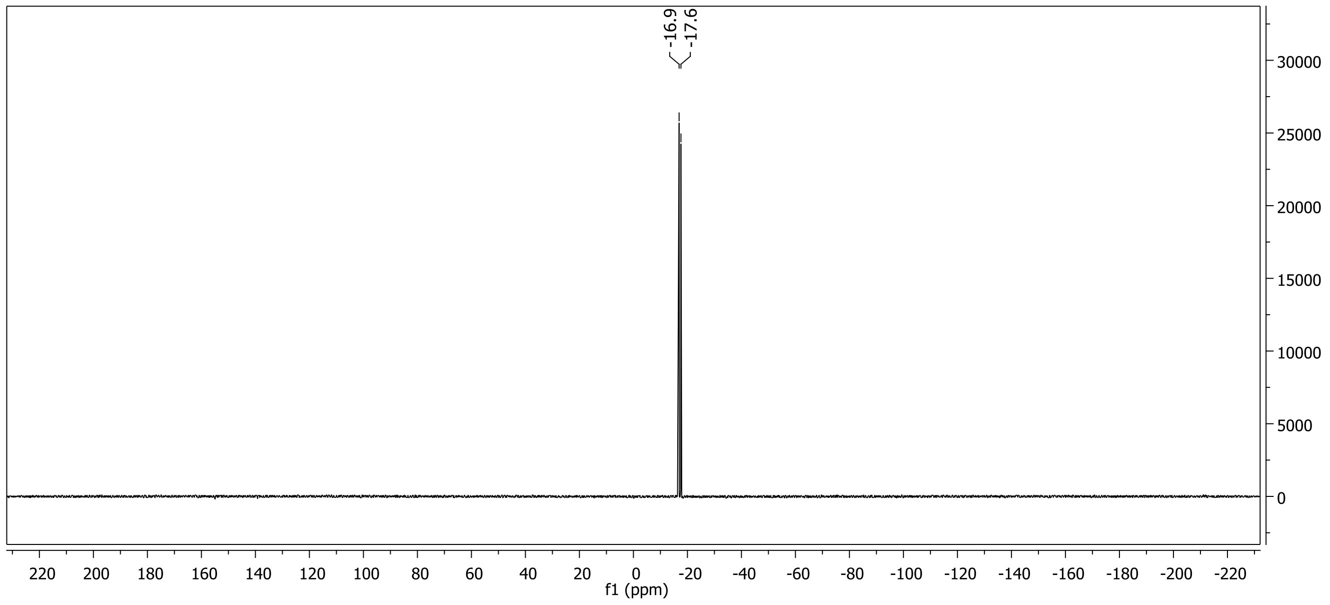

-16.9
-17.6
30000
25000
20000
15000
10000
5000
0
220 200 180 160 140 120 100 80 60 40 20 0 -20 -40 -60 -80 -100 -120 -140 -160 -180 -200 -220
f1 (ppm)

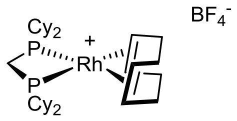

Cy2
P
Rh
P
Cy2
+
BF4-

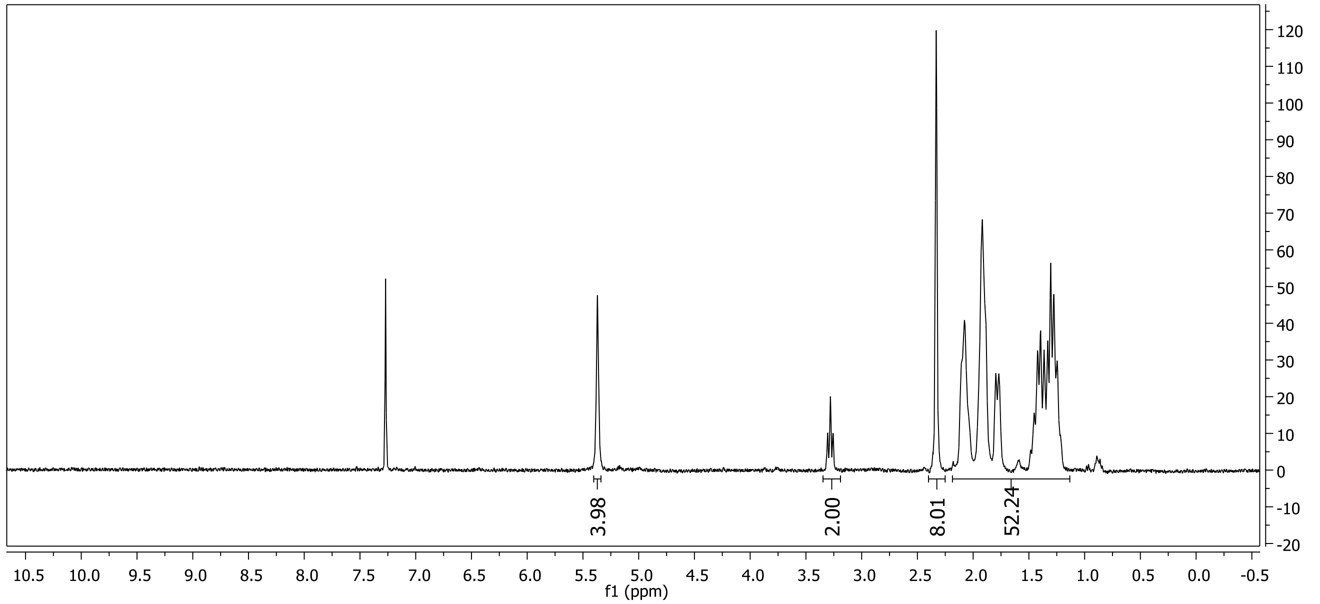

3.98
2.00
8.01
52.24
f1 (ppm)

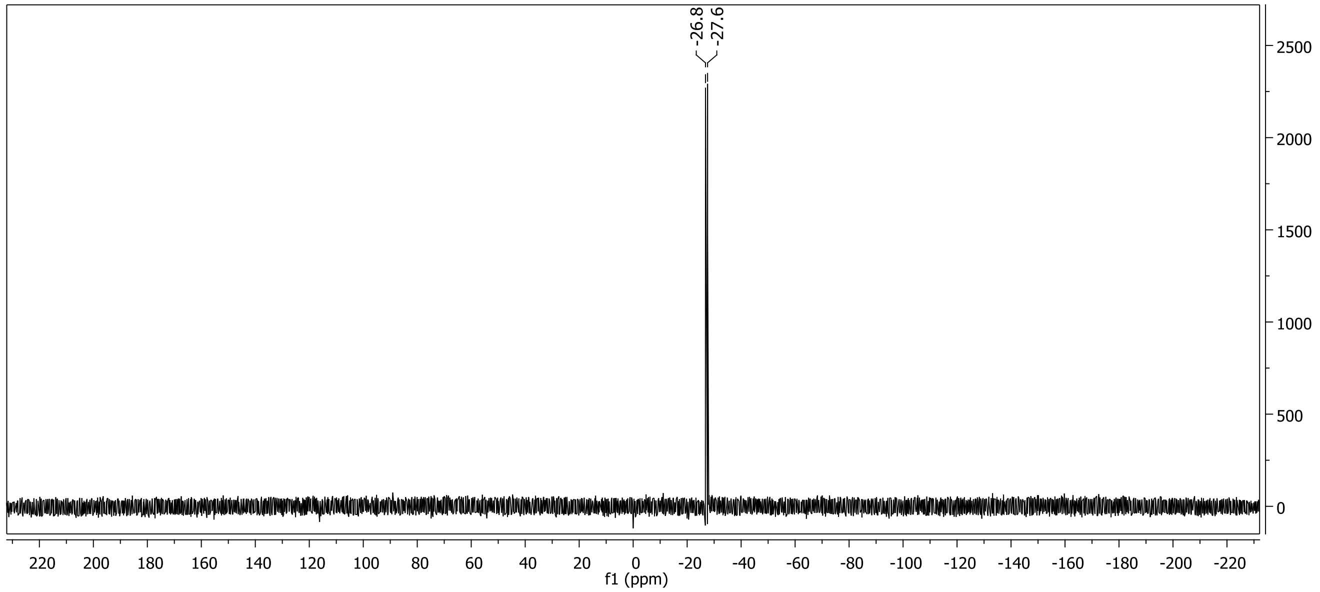

-26.8
-27.6
f1 (ppm)

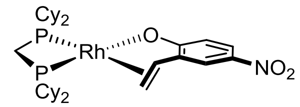

Cy2
P
Rh
O
NO2
P
Cy2

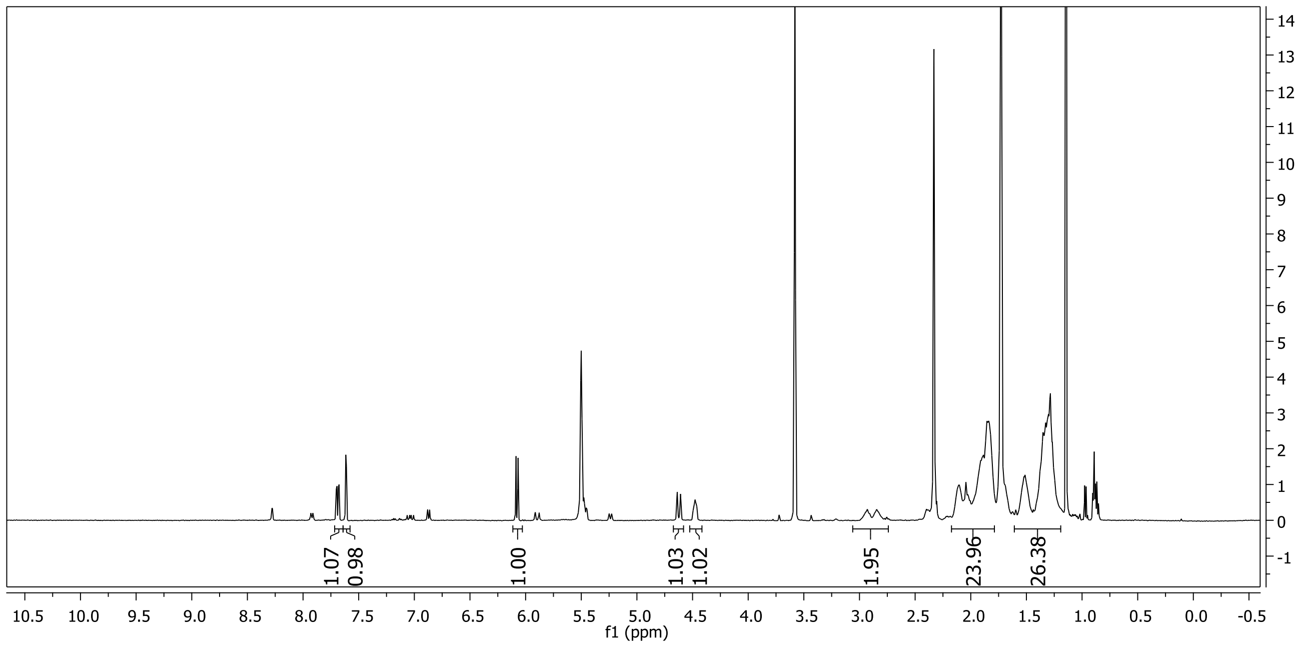

1.07
0.98
1.00
1.03
1.02
1.95
23.96
26.38
f1 (ppm)

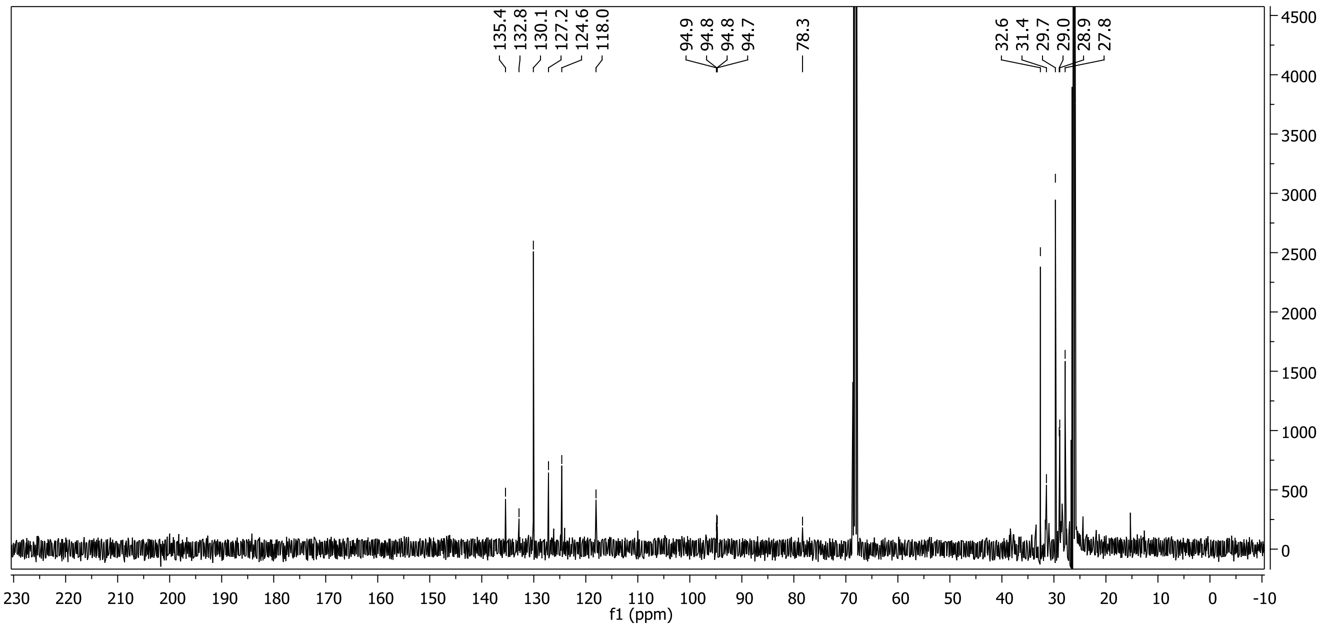

135.4
132.8
130.1
127.2
124.6
118.0
94.9
94.8
94.8
94.7
78.3
32.6
31.4
29.7
29.0
28.9
27.8
f1 (ppm)

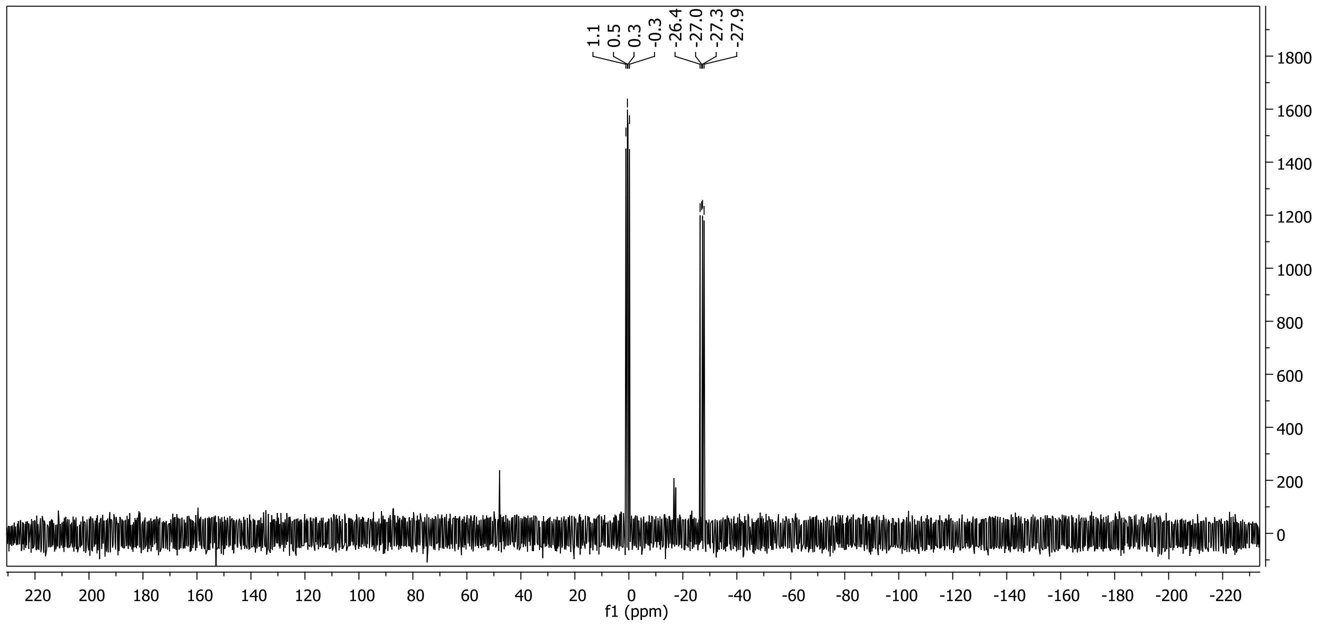
1.1
0.5
0.3
-0.3
-26.4
-27.0
-27.3
-27.9
1800
1600
1400
1200
1000
800
600
400
200
0
220
200
180
160
140
120
100
80
60
40
20
0
-20
-40
-60
-80
-100
-120
-140
-160
-180
-200
-220
f1 (ppm)

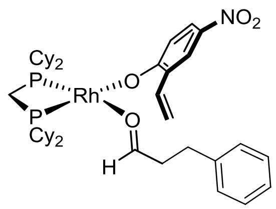

(from a reaction with excess aldehyde and olefin at steady state concentration, peaks in red brackets correspond to the title compound)

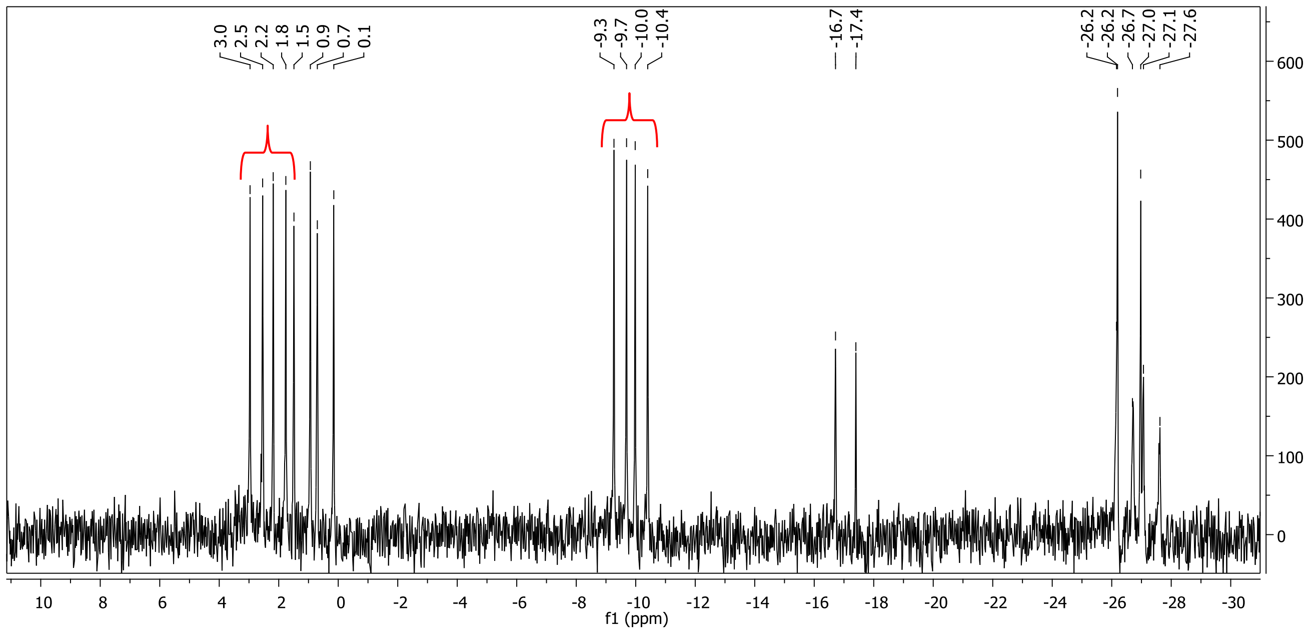

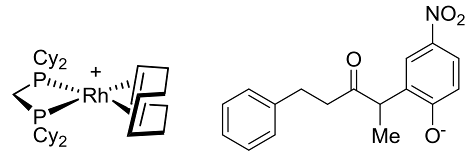

Cy2
P
Rh
P
Cy2
+
NO2
O
Me
O-

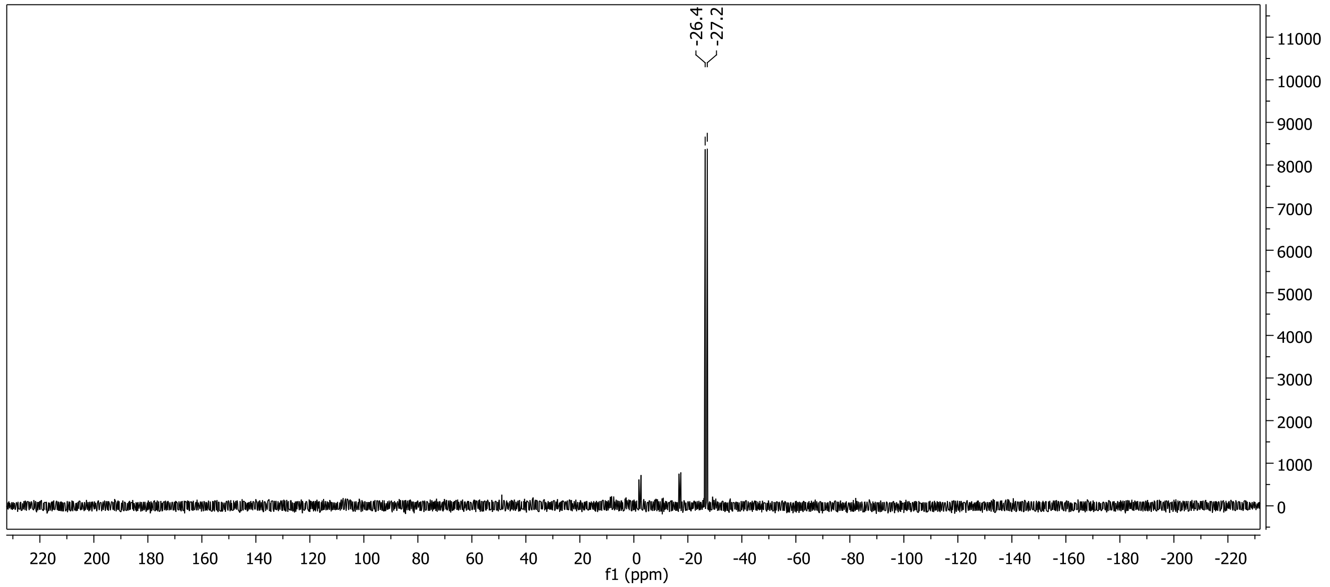

-26.4
-27.2
11000
10000
9000
8000
7000
6000
5000
4000
3000
2000
1000
0
220
200
180
160
140
120
100
80
60
40
20
0
-20
-40
-60
-80
-100
-120
-140
-160
-180
-200
-220
f1 (ppm)

8. Crystallographic Data

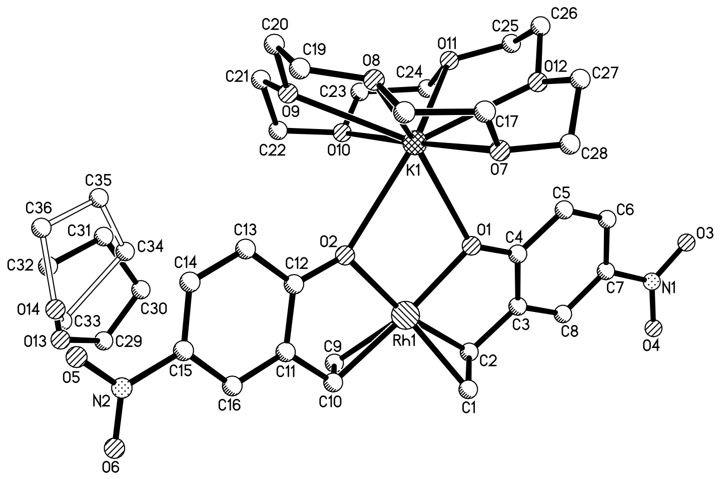

Table 1. Crystal data and structure refinement for vmd13.

Identification code	vmd13	
Empirical formula	$C_{28} H_{36} K N_2 O_{12} Rh \cdot C_4H_8O$	
Formula weight	806.70	
Temperature	88(2) K	
Wavelength	0.71073 Å	
Crystal system	Monoclinic	
Space group	*Cc*	
Unit cell dimensions	a = 13.6052(6) Å	a= 90°.
	b = 19.6523(9) Å	b= 105.5588(5)°.
	c = 14.3113(7) Å	g = 90°.
Volume	3686.2(3) Å^3	
Z	4	
Density (calculated)	1.454 Mg/m^3	
Absorption coefficient	0.640 mm^{-1}	
F(000)	1672	
Crystal color	yellow	
Crystal size	0.315 x 0.175 x 0.144 mm^3	
Theta range for data collection	1.868 to 27.103°	
Index ranges	$-17 \leq h \leq 17$, $-25 \leq k \leq 25$, $-18 \leq l \leq 18$	
Reflections collected	20977	

Independent reflections	8111 [R(int) = 0.0170]
Completeness to theta = 25.242°	99.9 %
Absorption correction	Numerical
Max. and min. transmission	0.9581 and 0.8741
Refinement method	Full-matrix least-squares on F^2
Data / restraints / parameters	8111 / 2 / 423
Goodness-of-fit on F^2	1.067
Final R indices [I>2sigma(I) = 7920 data]	R1 = 0.0258, wR2 = 0.0650
R indices (all data, 0.78Å)	R1 = 0.0267, wR2 = 0.0658
Absolute structure parameter	0.51(3)
Largest diff. peak and hole	0.550 and -0.336 e.$Å^{-3}$
